# 2D Layered Material Alloys: Synthesis and Application in Electronic and Optoelectronic Devices

**DOI:** 10.1002/advs.202103036

**Published:** 2021-10-31

**Authors:** Jiandong Yao, Guowei Yang

**Affiliations:** ^1^ State Key Laboratory of Optoelectronic Materials and Technologies Nanotechnology Research Center School of Materials Science & Engineering Sun Yat‐sen University Guangzhou Guangdong 510275 P. R. China

**Keywords:** 2D layered material alloys, alloy engineering, electronic devices, optoelectronic devices

## Abstract

2D layered materials (2DLMs) have come under the limelight of scientific and engineering research and broke new ground across a broad range of disciplines in the past decade. Nevertheless, the members of stoichiometric 2DLMs are relatively limited. This renders them incompetent to fulfill the multitudinous scenarios across the breadth of electronic and optoelectronic applications since the characteristics exhibited by a specific material are relatively monotonous and limited. Inspiringly, alloying of 2DLMs can markedly broaden the 2D family through composition modulation and it has ushered a whole new research domain: 2DLM alloy nano‐electronics and nano‐optoelectronics. This review begins with a comprehensive survey on synthetic technologies for the production of 2DLM alloys, which include chemical vapor transport, chemical vapor deposition, pulsed‐laser deposition, and molecular beam epitaxy, spanning their development, as well as, advantages and disadvantages. Then, the up‐to‐date advances of 2DLM alloys in electronic devices are summarized. Subsequently, the up‐to‐date advances of 2DLM alloys in optoelectronic devices are summarized. In the end, the ongoing challenges of this emerging field are highlighted and the future opportunities are envisioned, which aim to navigate the coming exploration and fully exert the pivotal role of 2DLMs toward the next generation of electronic and optoelectronic devices.

## Graphical abstract

A comprehensive overview on the synthesis of 2D layered material alloys, as well as, the corresponding electronic and optoelectronic devices is provided. In addition, the ongoing challenges are highlighted and the future opportunities are envisioned, which aim to navigate the coming exploration and fully exert the pivotal role of 2D materials toward the next generation of electronic and optoelectronic devices.

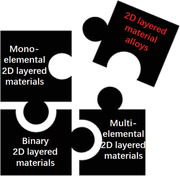

## 1 Introduction

The landmark cleavage of bulk graphite toward one‐atomic‐thick graphene in 2004,^[^ ^1^ ^]^ for which the Nobel Prize in Physics was awarded in 2010, accompanied with the exceptional electric and optoelectronic attributes,^[^ ^2^, ^3^ ^]^ have kicked off the prelude of flourishing research on 2D layered materials (2DLMs) across a broad range of disciplines.^[^ ^4^, ^5^, ^6^, ^7^, ^8^, ^9^, ^10^, ^11^, ^12^, ^13^, ^14^, ^15^, ^16^, ^17^ ^]^ In broad terms, 2DLMs represent the emerging atomically thin materials where intramolecular interactions are covalent/ionic forces, whereas the intermolecular interactions are van der Waals (vdW) forces. Inspiringly, the scientific communities have by far identified and produced a broad catalog of 2DLMs beyond graphene, represented by group 15 semiconductors,^[^ ^18^ ^]^ nitrides,^[^ ^7^ ^]^ transition metal dichalcogenides (TMDCs),^[^ ^19^, ^20^, ^21^, ^22^ ^]^ post‐transition metal chalcogenides,^[^ ^23^, ^24^ ^]^ halides,^[^ ^25^, ^26^, ^27^, ^28^ ^]^ multi‐elemental compounds,^[^ ^29^, ^30^, ^31^, ^32^, ^33^, ^34^, ^35^, ^36^ ^]^ just to name a few. Benefiting from the prominent electrostatic tunability, dangling‐bond‐free top/bottom interface, intrinsic exemption to short‐channel effects, spatially confined structures, reasonably high carrier mobility, outstanding mechanical strength, and strong interactions with light, these fascinating 2D building blocks have come under the limelight of the nano‐electronics and nano‐optoelectronics realms.^[^ ^37^, ^38^, ^39^, ^40^, ^41^ ^]^ Thus far, numerous accomplishments beyond the established norms have been effectuated. For example, in 2019, Zhang et al. demonstrated a field‐effect transistor (FET) with an effective gate length down to 4 nm based on a 2D MoTe_2_ channel.^[^ ^42^ ^]^ This device manifests prominent gate‐induced switching characteristic with an on/off ratio of ≈10^5^ and a subthreshold swing of 73 mV dec^−1^, revealing a broad prospect in the sub‐10 nm complimentary metal‐oxide‐semiconductor (CMOS) circuits. Recently, Maiti et al. have achieved a MoTe_2_ photodetector with a high responsivity of 0.5 A W^−1^ in the telecommunication C‐band (1550 nm) by leveraging strain engineering.^[^ ^43^ ^]^ Specifically, the introduction of ≈4% tensile strain in MoTe_2_ narrows its bandgap from 1.04 to 0.8 eV, which thus markedly broadens its effective wavelength range.

Despite having been extensively investigated from both experimental and theoretical aspects, the category of stoichiometric 2D layered materials (S2DLMs), namely, 2DLMs with a fixed atomic composition and structure, is yet far less than that of conventional semiconductors. This renders them incompetent to fully satisfy the multitudinous scenarios across the entire breadth of electronic and optoelectronic applications, and it's thereby challenging for 2DLMs to take the place of traditional semiconductors. By virtue of the added degree of freedom in composition, alloying, a strategy widely exploited in conventional semiconductors,^[^ ^44^ ^]^ has been identified as a powerful strategy to tailor the electric and optoelectronic attributes in a wide range without breaking the structural integrity.^[^ ^45^ ^]^ To begin with, alloying semiconductors with distinct charge‐carrier polarities enables the modulation of electric transport properties. In addition, alloying represents a flexible route to tune the energy band structure of semiconductors, which can thus be exploited to tailor the optical properties. For example, as the As content increases from 0% to 100%, the peak position of the photoluminescence (PL) spectrum of the InAs*_x_* P_1−_ *_x_* alloy continuously evolves from 860 to 3070 nm.^[^ ^46^ ^]^ Similarly, mercury cadmium telluride alloy with composition‐dependent direct bandgap has been developed for mid‐wave infrared to long‐wave infrared (3–15 µm) photodetection.^[^ ^47^ ^]^ Such wide‐range modulation of bandgap far exceeds other strategies such as quantum confinement,^[^ ^48^ ^]^ strain engineering,^[^ ^49^ ^]^ and regulation of dielectric environment.^[^ ^50^ ^]^ In this regard, alloying facilitates on‐demand design of exotic semiconductors according to the down‐to‐earth electronic and optoelectronic applications.

Inspiringly, numerous theoretical investigations have prefigured that alloying can markedly expand the library of 2DLMs as well and it will bring a variety of exotic physical properties beyond the fundamental limits of S2DLMs. For example, in 2015, Huang et al. predicted that the deep‐level defect states can be markedly suppressed in 2D Mo_1−_ *_x_* W*_x_* Se_2_ alloy with a low W content.^[^ ^51^ ^]^ In 2019, Zhang et al. predicted that the energy band alignment and energy band offset of the 2D Mo_1−_ *_x_* W*_x_* Y_2_ (Y = S, Se) alloy based heterojunction can be readily modulated by tailoring the composition of the alloy.^[^ ^52^ ^]^ In particular, a remarkable photoelectric power conversion efficiency of up to 23.4% can be achieved based on the optimally designed heterostructure. It is noteworthy that a number of 2DLMs share a common stoichiometric formula (e.g., MX,^[^ ^53^, ^54^, ^55^, ^56^, ^57^, ^58^, ^59^ ^]^ MX_2_,^[^ ^60^, ^61^, ^62^, ^63^, ^64^, ^65^, ^66^, ^67^, ^68^, ^69^, ^70^ ^]^ MX_3_,^[^ ^71^, ^72^, ^73^, ^74^ ^]^ M_2_X_3_,^[^ ^75^, ^76^, ^77^, ^78^, ^79^, ^80^ ^]^ ABX_2_,^[^ ^81^, ^82^ ^]^ ABX_3_,^[^ ^35^, ^83^, ^84^, ^85^ ^]^ etc.) and analogous crystal structures. Such high similarities promise a high miscibility, that is, the complete alloying across the whole composition range without compromising the crystalline quality. In this regard, theoretically innumerable 2DLM alloys are available to tie in with variable scenarios in the integrated electronic and optoelectronic applications.

Thus far, researchers have made considerable progress in the synthesis of 2DLM alloys as well. For example, Li et al. have reported on a facile one‐step chemical vapor deposition (CVD) growth of 2D MoS_2_ *_x_* Se_2(1−_ *_x_*_)_ alloy with complete composition tunability (0 ≤ *x* ≤ 1).^[^ ^86^ ^]^ In the wake of the prodigious accomplishments in material synthesis, a series of distinctive electronic and optoelectronic attributes of 2DLM alloys have been unveiled. In 2017, Rhodes et al. revealed a fascinating semiconductor to semi‐metal transition in 2D MoSe_2_ *_x_* Te_2(1−_ *_x_*_)_ alloy with increasing Te content.^[^ ^87^ ^]^ In another work, Ernandes et al. found that WS_0.4_Se_1.6_ alloy exhibited a strong immunity to PL quenching with increasing thickness as compared to the binary WS_2_ and WSe_2_.^[^ ^88^ ^]^ Most recently, Guo et al. have achieved a high‐performance phototransistor based on a MoSe_2_ *_x_* Te_2−2_ *_x_* thin layer.^[^ ^89^ ^]^ This device demonstrates a pronounced photoswitching characteristic with an on/off ratio of ≈10^5^. Furthermore, 2DLM alloys have also been exploited for vdW heterostructures. As the Se content increases from 0% to 100%, the band structure of a PbI_2_/WS_2(1−_ *_x_*_)_Se_2_ *_x_* heterojunction will transit from straddling type‐I alignment to staggered type‐II alignment.^[^ ^90^ ^]^ As a consequence, the PL of WS_2(1−_ *_x_*_)_Se_2_ *_x_* can be dramatically modulated from apparently enhanced to greatly quenched as compared to an independent monolayer WS_2(1−_ *_x_*_)_Se_2_ *_x_* counterpart. Beyond that, 2DLM alloys have also proven as compelling building blocks toward fundamental physics and novel discoveries. For example, it is found that the valley splitting of Co*_x_* Mo_1−_ *_x_* S_2_ can be markedly boosted by more than 100% as compared to pristine MoS_2_.^[^ ^91^ ^]^ On the whole, alloying can break the intrinsic limitations of S2DLMs and it has opened up unexpected and abundant opportunities to the design and implementation of exotic material properties and device functionalities.

Despite tremendous advancements, the studies on 2DLM alloy electronic and optoelectronic devices are scattered, and the underlying fundamentals are elusive. In addition, the published reviews on 2D devices have been mostly dedicated to S2DLMs.^[^ ^14^, ^40^, ^92^, ^93^, ^94^, ^95^, ^96^, ^97^ ^]^ To our knowledge, a timely and comprehensive overview on both the production of 2DLM alloys as well as their application in electronic and optoelectronic nanodevices is yet to be provided, despite it is probably a critical step toward navigating further exploration on this nascent field. To this end, this review begins with a comprehensive summary on various methods for the production of 2DLM alloys, including chemical vapor transport (CVT), CVD, pulsed‐laser deposition (PLD), and molecular beam epitaxy (MBE). Then, the application of 2DLM alloys in electronic devices is summarized. Subsequently, the application of 2DLM alloys in optoelectronic devices is summarized. In the end, the ongoing challenges of this rapidly progressing domain are highlighted and the potential schemes to address them are envisioned, which aim to navigate and motivate the future research and fully exert the pivotal role of 2DLMs toward the next generation of electronic and optoelectronic devices.

## 2 Production of 2D Layered Material Alloys

In contrast to the multiple stoichiometric layered materials naturally occurring in minerals,^[^ ^98^ ^]^ there are few natural alloyed counterparts. Therefore, the production of high‐quality 2DLM alloys is of fundamental importance for the in‐depth exploration of the fundamental physics and device demonstration. However, compared to S2DLMs, the growth of 2DLM alloys is much more technically challenging since the incorporation of heteroatoms may induce lattice distortions, and it will make the growth kinetics much more complicated. Thus far, extensive research enthusiasms have been dedicated to the production of 2DLM alloys for both electronic and optoelectronic applications. Inspiringly, significant accomplishments have been achieved based on a series of growth techniques, including CVT, CVD, PLD, and MBE. Each of these techniques manifests its own advantages and disadvantages, and they will be critically introduced in detail below. It is noteworthy that a series of solution‐processed synthesis methods, such as, hydrothermal reaction,^[^ ^99^, ^100^, ^101^ ^]^ thermolysis,^[^ ^102^ ^]^ Schlenk line synthesis,^[^ ^103^ ^]^ and liquid exfoliation,^[^ ^104^, ^105^ ^]^ suffer from insurmountable contamination issues (e.g., dispersants, residuals and by‐products), low crystalline quality of products and poor compatibility with the state‐of‐the‐art micro‐fabrication technology, which severely hamper their application in the field of electronic and optoelectronic devices. Therefore, these approaches have not been included in this review.

### 2.1 Chemical Vapor Transport

**Figure 1:**
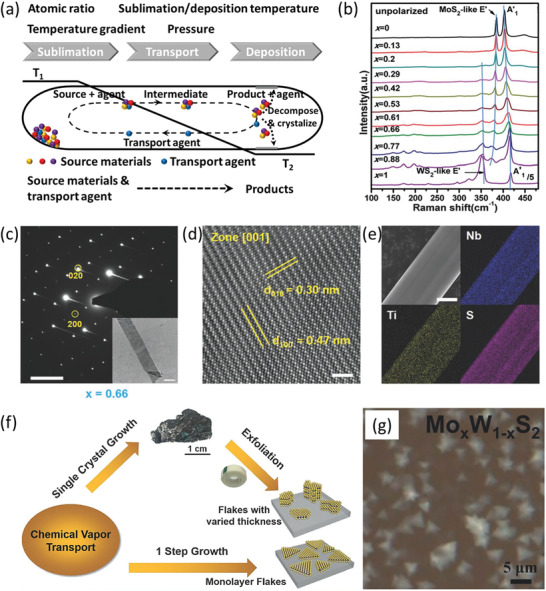
CVT growth of 2DLM alloys. a) Schematic diagram of the typical experimental setup of CVT growth of layered material alloys and the affecting factors. b) Raman spectra of the CVT‐grown Mo_1−_ *_x_* W*_x_* S_2_ alloys with different compositions.^[^ ^126^ ^]^ Reproduced with permission.^[^ ^126^ ^]^ Copyright 2014, Royal Society of Chemistry. c) Selected area electron diffraction pattern, d) high‐resolution transmission electron microscope image and e) element mapping images of the CVT‐grown Nb_1−_ *_x_* Ti*_x_* S_3_ alloy.^[^ ^125^ ^]^ c–e) Reproduced with permission.^[^ ^125^ ^]^ Copyright 2020, John Wiley and Sons, Inc. f) Schematic illustration of two routes for producing 2DLMs via CVT. g) An optical microscope image of the as‐grown few‐layer Mo*_x_* W_1−_ *_x_* S_2_.^[^ ^127^ ^]^ f–g) Reproduced with permission.^[^ ^127^ ^]^ Copyright 2017, John Wiley and Sons, Inc.

CVT, a mature and powerful production technique for the crystallization of variable compositions, represents a category of reactions where source materials are generally evaporated/sublimated from a hot side and finally crystallize at a cold side via a sublimation‐transport‐solidification process.^[^ ^106^ ^]^ A typical experimental configuration of CVT is presented in **Figure** **1**a. Basically, thoroughly mixed proportional source materials and transport agents (mineralizers) under vacuum condition are heated to produce the gaseous intermediates. As a consequence, a partial pressure gradient of the gaseous intermediates is generated, which drives them to transport from the high concentration region to the low concentration region. Then, these gaseous species decompose and deposit at the cold end in the form of crystals whilst releasing the transport agent. Consequently, the partial pressure of transport agent at the deposition region increases, which thus drives them to transport back to the source material side for the subsequent reaction. Taking the synthesis of SnSe_2(1−_ *_x_*_)_S_2_ *_x_* alloy for example,^[^ ^107^, ^108^ ^]^ Sn, S, and Se powders with the nominal atomic molar ratio are used as source materials, and a small amount of I_2_ powder is used as transport agent. Specifically, mixed Sn, S, Se, and I_2_ powders are loaded into a quartz ampoule, after which the ampoule is evacuated down to a low pressure and sealed to serve as a closed reaction chamber. Then, the ampoule is horizontally placed in a furnace with a temperature gradient between its two ends, where the source materials are posited at the hot end and the colder end is the deposition zone. In the growth process, the source materials react with the transport agent to form volatile intermediates through a reversible endothermic reaction. Then, these species transport to the cold end, where they decompose and crystallize into monocrystalline SnSe_2(1−_ *_x_*_)_S_2_ *_x_* alloy.

Thus far, CVT has been successfully exploited to synthesize a host of layered material alloys such as Mo_1−_ *_x_* W*_x_* S_2_,^[^ ^109^, ^110^ ^]^ Mo_1−_ *_x_* Nb*_x_* Se_2_,^[^ ^111^ ^]^ Nb*_x_* Re_1−_ *_x_* Se_2_,^[^ ^112^ ^]^ MoSe_2_ *_x_* Te_2(1−_ *_x_*_)_,^[^ ^89^, ^113^ ^]^ WSe_2(1−_ *_x_*_)_Te_2_ *_x_*,^[^ ^114^ ^]^ ZrS*_x_* Se_2−_ *_x_*,^[^ ^115^ ^]^ HfS*_x_* Se_2−_ *_x_*,^[^ ^116^ ^]^ HfS_2(1−_ *_x_*_)_Te_2_ *_x_*,^[^ ^117^ ^]^ TaSe_2−_ *_x_* S*_x_*,^[^ ^118^ ^]^ Sn(S*_x_* Se_1−_ *_x_*)_2_,^[^ ^119^ ^]^ In*_x_* Sn_1−_ *_x_* S_2_,^[^ ^120^ ^]^ V*_x_* Sn_1−_ *_x_* S_2_,^[^ ^121^ ^]^ Pb*_x_* Sn_1−_ *_x_* Se_2_,^[^ ^122^ ^]^ Ga_1−_ *_x_* In*_x_* Se,^[^ ^123^ ^]^ MnPS_3−_ *_x_* Se*_x_* ^[^ ^124^ ^]^ and Nb_1−_ *_x_* Ti*_x_* S_3_,^[^ ^125^ ^]^ just to name a few, demonstrating the broad generalizability of this approach. In addition, the composition of the CVT‐derived alloys can be facilely customized in a wide range, since the source materials are sealed in an enclosed environment, which can effectively prevent the unintentional volatilization. For example, Chen et al. have produced a series of monolayer Mo_1−_ *_x_* W*_x_* S_2_ alloys with the W content ranging from 0% to 100% from the corresponding CVT‐grown bulk crystals (Figure 1b).^[^ ^126^ ^]^ The vacuumed and sealed reaction chamber accompanied with the precisely controlled compositions can also give rise to high cleanness and repeatability. Furthermore, by virtue of the long‐term annealing process at a high reaction temperature that provides sufficient time and energy for the crystallization and complete alloying of the components, the CVT‐derived alloys possess high crystal quality, normally in a single crystal form, and homogeneous element distribution (Figure 1c–e). Beyond that, compared to PLD and MBE, CVT is cost‐efficient as no sophisticated equipment and high‐cost source materials are required.

Nevertheless, CVT has still been stuck with some deficiencies that preclude its widespread application in the field of 2DLM alloy electronic and optoelectronic devices. First, the alloys produced by CVT are generally bulk crystals. Therefore, an additional micromechanical exfoliation procedure, which is not scalable, is required to produce the ultrathin 2D counterparts. It is rather labor‐intensive and time‐consuming, making CVT unavailable for large‐scale application. Importantly, by reducing the amount of metal sources and transport agent whilst increasing the temperature gradient between the reaction and deposition sides, the direct growth of 2DLMs onto targeted substrates has been achieved (Figure 1f,g).^[^ ^127^ ^]^ However, this is at the expense of dramatically suppressed growth rate. Another primary challenge is that the lateral domain size of both the exfoliated and the directly grown samples is generally limited to tens of micrometers. This makes them suffer from severe large‐scale integration difficulties. Finally, CVT requires an extremely long growth period (normally tens of hours and beyond) under high‐temperature conditions, which is adverse to the direct back‐end‐of‐line integration regarding the strict thermal restriction of substrates. As a consequence, the CVT‐derived 2DLM alloys have been largely limited to fundamental studies in the current stage.

### 2.2 Chemical Vapor Deposition

**Figure 2:**
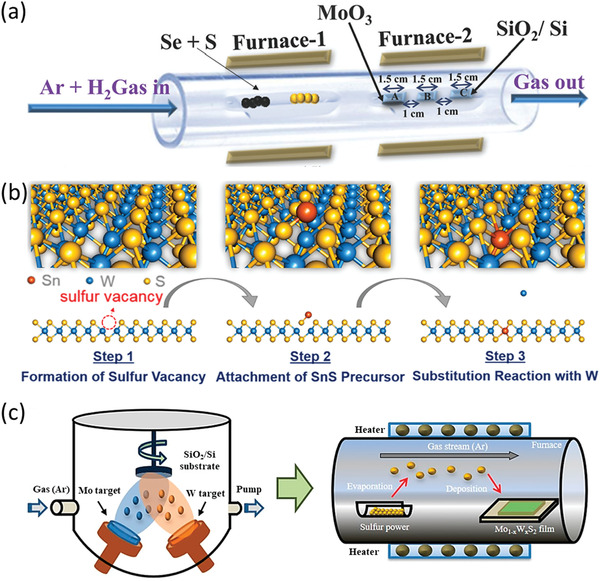
CVD growth of 2DLM alloys. a) Schematic diagram of the CVD growth of 2D MoS_2(1−_ *_x_*_)_Se_2_ *_x_* alloy.^[^ ^130^ ^]^ Reproduced with permission.^[^ ^130^ ^]^ Copyright 2017, Royal Society of Chemistry. b) Schematic illustration of the mechanism for a two‐step CVD growth of 2D Sn*_x_* W_1−_ *_x_* S_2_ alloy.^[^ ^132^ ^]^ Reproduced with permission.^[^ ^132^ ^]^ Copyright 2019, American Chemical Society. c) Schematic illustration of a two‐step synthesis procedure of 2D Mo_1−_ *_x_* W*_x_* S_2_ alloy.^[^ ^133^ ^]^ Reproduced with permission.^[^ ^133^ ^]^ Copyright 2018, American Chemical Society.

CVD has been appraised as one of the most extensively explored bottom‐up approaches for the production of 2DLMs.^[^ ^128^, ^129^ ^]^ Generally, for the synthesis of 2DLM alloys, multiple precursors containing various components are required to be exploited simultaneously. Taking the synthesis of 2D MoS_2(1−_ *_x_*_)_Se_2_ *_x_* alloy for example, S and Se powders are used as the chalcogen sources, while MoO_3_ powder is used as the metal source (**Figure** **2**a).^[^ ^130^ ^]^ Specifically, S and Se powders are located at the upstream heating zone and MoO_3_ powder is located at the downstream heating zone. The SiO_2_/Si substrates are set to be facing down on top of the MoO_3_ boat for collection/deposition of products. As the temperature of the furnaces elevates, the precursors sublimate. The gaseous chalcogen species transit from upstream to downstream with the assist of a mixed Ar and H_2_ gas flow. Finally, these species reach the SiO_2_/Si substrates and then crystalize into 2D nanosheets atop. Similarly, Zou et al. produced 2D V*_x_* Mo_1−_ *_x_* S_2_ nanosheets by exploiting V_2_O_5_/NH_4_VO_3_/VCl_3_ and MoO_3_ powders as metal precursors as well as S powder as chalcogen precursor.^[^ ^131^ ^]^ Zhou et al. synthesized Co*_x_* Mo_1−_ *_x_* S_2_ nanosheets by using CoCl_2_ and MoO_3_ powders as metal precursors as well as S powder as chalcogen precursor.^[^ ^91^ ^]^ In addition to the direct synthesis by synchronously evaporating various components, 2DLM alloys can be achieved by post‐synthesis incorporation of heteroatoms into the S2DLM matrix as well. Recently, Chang et al. developed a two‐step process for the preparation of 2D Sn*_x_* W_1−_ *_x_* S_2_ alloy (Figure 2b).^[^ ^132^ ^]^ To begin with, 2D WS_2_ monolayers are synthesized via CVD. Then, a post‐growth thermal doping process is conducted by annealing the WS_2_ monolayers under a Sn‐rich environment, which is afforded by heating the SnS precursor.

On the whole, CVD also manifests broad generalizability for the growth of 2DLM alloys. For example, Zhou et al. have synthesized 13 kinds of 2DLM alloys by exploiting a single molten‐salt‐assisted CVD approach.^[^ ^134^ ^]^ In addition, taking advantages of the high growth temperature enabling sufficient crystallization, the CVD‐derived products possess relatively high crystallinity, which is conducive to device demonstration. Furthermore, CVD is economic and efficient as it can commonly be performed under low‐vacuum environment and even under atmospheric pressure. Despite significant advancements, there are still several issues to be addressed prior to the practical application of CVD in industrial production of 2DLM alloys. First, different reaction precursors normally have discrepant thermal properties. For example, the melting points of MoO_3_ and WO_3_ are ≈795 °C and ≈1473 °C, respectively. The large discordance may result in asynchronous sublimation of precursors, and thereby poor controllability of the atomic molar ratio, inhomogeneous element distribution and even phase segregation of the products provided the growth conditions are not optimized.^[^ ^135^, ^136^, ^137^, ^138^ ^]^ Second, in spite of the excellent scalability of CVD, the lateral domain size of the CVD‐derived 2DLM alloys is normally limited to tens of micrometers,^[^ ^139^, ^140^, ^141^, ^142^ ^]^ which is unqualified for realizing large‐scale integration. To address these issues, several approaches have been developed. For example, Park et al. synthesized wafer‐scale 2D Mo_1−_ *_x_* W*_x_* S_2_ alloy with uniform composition distribution through co‐sputtering of a Mo_1−_ *_x_* W*_x_* metal film followed by sulfurization treatment to transform it into metal dichalcogenides (Figure 2c).^[^ ^133^ ^]^ In addition, molten‐salt‐assisted CVD may provide a flexible route to match the volatilization of various precursors,^[^ ^143^ ^]^ which will be in need of in‐depth investigation in the future. On the other hand, Zhang et al. have developed a confined‐space CVD approach for the production of large‐size 2DLM alloys.^[^ ^144^ ^]^ Specifically, two vertically stacked SiO_2_/Si substrates are employed during the material growth: One for the deposition of 2DLM alloys, and the other acting as an assistant substrate for space confinement. Importantly, Mo_1−_ *_x_* W*_x_* S_2_ monolayers with an average lateral size up to 300 µm and a maximum size up to 500 µm are achieved. This is ascribed to the protection effect of the assistant substrate, which promises a stable and clean growth environment markedly suppressing the nucleation. Similarly, Kang et al. successfully synthesized ReS_2(1−_ *_x_*_)_Se_2_ *_x_* monolayers with a lateral size up to 200 µm through a salt‐assisted confined‐space CVD approach.^[^ ^145^ ^]^

Third, CVD growth is generally performed under high temperature, which is normally higher than 900 K.^[^ ^130^, ^146^, ^147^, ^148^ ^]^ This makes it suffer from a considerably large thermal budget and low degree of compatibility with the commercially available flexible substrates such as polymide, polydimethylsiloxane and polyethylene terephthalate, which is the precondition for top‐surface integration. This severely hampers the development of CVD for the construction of wearable devices. Fourth, it is noteworthy that there are a series of parameters, including but not limited to the precursors, substrates, gas atmosphere, gas flow rate, growth temperature, heating/cooling rate, location of precursors/substrates, and heating duration, that play a critical role in the morphology, orientation, composition, thickness, and crystalline quality of products. The parameter space for the CVD growth of a specific 2DLM alloy is rather narrow. Restricted by the rough controllability in the growth dynamics and the nonuniform supply of precursors, the repeatability of CVD is relatively poor and the products are sometimes messy (e.g., position‐dependent nucleation and morphology). For example, in the CVD growth of 2D Mo_1−_ *_x_* W*_x_* S_2_ alloy by Wang et al., monolayer and multilayer Mo_1−_ *_x_* W*_x_* S_2_ nanosheets are randomly distributed throughout the substrates.^[^ ^141^ ^]^ In addition, contaminations and crystal defects will be inevitably induced by the precursors. For example, the use of metal oxide precursors for the growth of TMDCs may result in the formation of O‐passivated chalcogen vacancies and O interstitials.^[^ ^149^ ^]^

### 2.3 Pulsed‐Laser Deposition

PLD is a high‐vacuum physical vapor deposition technique that reproduces the composition of targets onto the intended substrates with the assisted of pulsed laser.^[^ ^37^, ^150^, ^151^ ^]^ Basically, a focused pulsed laser beam is directed onto the surface of a target, which bombards the surface layer and ejects plasma plumes containing a myriad of species stoichiometrically proportional to the target in an ultra‐short time scale (normally down to tens of nanoseconds). These highly active species then transport downstream and adhere to the surface of the targeted substrates, which are normally set to be opposite to the plasma plumes. Finally, they recrystallize, usually in a 2D thin film form, through a complicated process of diffusion, nucleation, crystallization and growth. Since the focused pulsed laser beam possesses high energy flux, the composition of targets can be non‐selectively and synchronously bombarded, which warrants stable and highly controlled growth conditions. In this regard, compared to other growth techniques, PLD holds the intrinsic benefits for the production of multi‐component compounds with complicated phase diagrams,^[^ ^152^, ^153^, ^154^ ^]^ making it a powerful growth technique for synthesizing 2DLM alloys. Actually, 2D Mo_0.5_W_0.5_S_2_,^[^ ^155^ ^]^ Mo_0.5_W_0.5_Se_2_,^[^ ^156^ ^]^ Sn_1−_ *_x_* As*_x_* Se,^[^ ^157^ ^]^ Bi_2_Te_2.7_Se_0.3_,^[^ ^158^, ^159^, ^160^ ^]^ and BiInSe_3_^[^ ^161^ ^]^ alloys have been experimentally synthesized through PLD.

**Figure 3:**
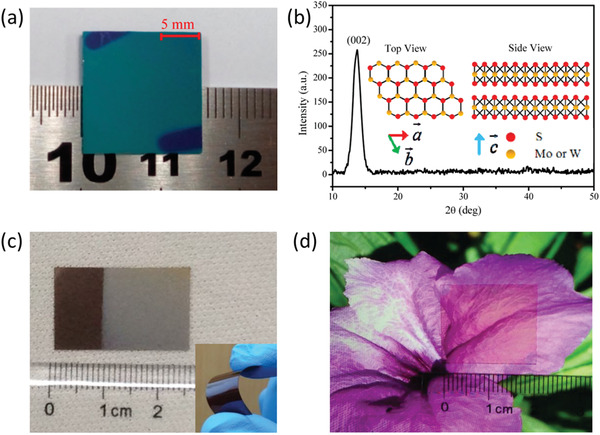
PLD growth of 2DLM alloys. a) Digital photograph of the PLD‐grown Mo_0.5_W_0.5_S_2_ alloy on a SiO_2_/Si substrate. b) X‐ray diffraction pattern. The inset shows the schematic diagrams of the crystal structure.^[^ ^155^ ^]^ a–b) Reproduced with permission.^[^ ^155^ ^]^ Copyright 2016, American Chemical Society. c,d) Digital photographs of the PLD‐grown Mo_0.5_W_0.5_S_2_ alloy on polyimide and sapphire substrates.^[^ ^156^ ^]^ c,d) Reproduced with permission.^[^ ^156^ ^]^ Copyright 2017, American Chemical Society.

Importantly, PLD manifests a host of merits distinguishing it from other growth techniques. To begin with, conforming to the emanative plasma plumes, PLD is highly scalable,^[^ ^162^ ^]^ where the lateral size of products is generally limited by the size of substrates. Inspiringly, a series of centimeter‐scale, continuous and uniform 2DLM alloys have been produced via PLD. For example, in 2016, Yao et al. synthesized highly c‐axis oriented multilayer Mo_0.5_W_0.5_S_2_ alloy with uniform composition distribution over a 1.5 × 1.5 cm^2^ SiO_2_/Si substrate (**Figure** **3**a,b).^[^ ^155^ ^]^ The large‐scale preparation is of fundamental importance toward harnessing the appealing attributes of 2DLM alloys in practical electronic and optoelectronic applications. In addition, the thickness of PLD‐derived products can be intentionally tuned by conveniently modulating the pulse number, laser energy, substrate–target distance, etc., a distinctive superiority over CVT and CVD. Beyond that, since the compositions of the PLD‐derived 2DLMs are approximately in accordance with the ablated source materials, the atomic molar ratio of the PLD‐grown 2DLM alloys can be readily controlled by expediently modulating the atomic molar ratio of the corresponding bulk targets. It endows PLD with much superior repeatability as compared to CVD as for synthesizing pre‐determined 2DLM alloys. Finally, since the plasma species bombarded by high‐energy focused pulsed laser are energetic, the growth temperature of PLD is markedly lowered as compared to that of CVD.^[^ ^163^, ^164^ ^]^ It endows PLD with a high degree of compatibility with various substrates, notably the polymer ones unsustainable to high temperature. For example, by using PLD, Zheng et al. have effectuated the direct deposition of 2D Mo_0.5_W_0.5_Se_2_ alloy on both polymide and Al_2_O_3_ substrates under a substrate temperature of merely 405 °C (Figure 3c,d),^[^ ^156^ ^]^ which is ≈500 °C lower than the substrate temperature demanded for the CVD growth of Mo_0.5_W_0.5_Se_2_.^[^ ^165^ ^]^ The low growth temperature also makes PLD much more compatible with the direct back‐end‐of‐line integration.

In spite of numerous advantages, PLD suffers from relatively high production cost as compared to CVD since a high‐vacuum growth environment is demanded. In addition, PLD‐derived 2DLMs are generally polycrystalline, and there are thus abundant crystallites/grain boundaries.^[^ ^166^, ^167^, ^168^ ^]^ Moreover, the surface morphology of the PLD‐grown 2DLMs is relatively rough as compared to that of the CVD‐grown and mechanically exfoliated samples. Both of these factors are inconducive to carrier transport. Therefore, further optimization of the growth parameters (e.g., the gas atmosphere, partial pressures of gases, substrate temperature, target structure, target composition, laser energy, etc.)^[^ ^37^ ^]^ and post‐growth treatments are in need of development to address these issues. Recently, Xie et al. have demonstrated that annealing of the PLD‐grown chalcogenides in a chalcogen‐rich atmosphere is a promising route to eliminate the oxides and chalcogen vacancies.^[^ ^169^ ^]^ Theoretically, this strategy can be generally applicable across various 2DLM platforms, making it a versatile scheme to ameliorate the crystalline quality of 2DLM alloys in the future.

### 2.4 Molecular Beam Epitaxy

**Figure 4:**
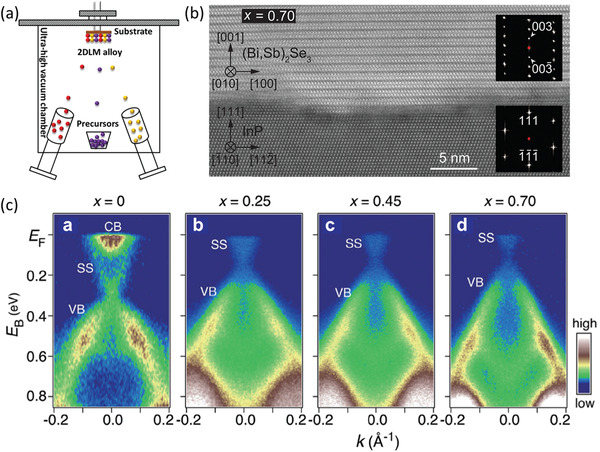
MBE growth of 2DLM alloys. a) Schematic diagram of a typical MBE growth system. b) High‐resolution scanning transmission electron microscope image of the MBE‐grown 2D (Bi_1−_ *_x_* Sb*_x_*)_2_Se_3_ alloy. The top and bottom insets show the corresponding selected area electron diffraction patterns from the (Bi_1−_ *_x_* Sb*_x_*)_2_Se_3_ and substrate, respectively. c) Angle‐resolved photoemission spectroscopy images of the (Bi_1−_ *_x_* Sb*_x_*)_2_Se_3_ alloys for *x* = 0 c_a_), 0.25 c_b_), 0.45 c_c_), 0.7 c_d_).^[^ ^172^ ^]^ b,c) Reproduced with permission.^[^ ^172^ ^]^ Copyright 2018, IOP Publishing Ltd.

MBE is a sophisticated layer‐by‐layer vapor deposition technique proceeded in an ultra‐high vacuum environment (normally with a base vacuum better than 1 × 10^−9^ mbar), where the highly pure reaction precursors are fed through the thermal sublimation or electron beam evaporation of the corresponding source materials (**Figure** **4**a). Taking the synthesis of Mo_1−_ *_x_* W*_x_* Se_2_ alloy for example,^[^ ^170^ ^]^ Mo flux and W flux are produced by using electron‐beam heating evaporators, while Se (99.9995%) is sublimated from a standard Knudsen cell. These precursors then transport downstream and attach to the surface of targeted substrates, which are set to be opposite to the sources, finally crystallizing into 2D Mo_1−_ *_x_* W*_x_* Se_2_ alloy.

Compared with the above growth techniques, MBE manifests a series of peculiar advantages. To begin with, MBE is easily scalable and versatile for the growth of various 2DLM alloys. Thus far, it has been successfully exploited to synthesize 2D (Bi_1−_ *_x_* Sb*_x_*)_2_Te_3_,^[^ ^171^ ^]^ (Bi_1−_ *_x_* Sb*_x_*)_2_Se_3_,^[^ ^172^ ^]^ GaTe*_x_* Se_1−_ *_x_*,^[^ ^173^ ^]^ WSe_2−_ *_x_* Te*_x_*,^[^ ^174^ ^]^ Mo*_x_* W_1_ *_−x_* Se_2_,^[^ ^170^ ^]^ V*_x_* Mo_1_ *_−x_* Se_2_,^[^ ^175^ ^]^ and As*_x_* Te_1_ *_−x_* ^[^ ^176^ ^]^ alloys.

In addition, by virtue of the precisely modulated supply rate of the molecular beam flux, the thickness of MBE‐grown 2DLMs can be readily controlled. As a result, the repeatability is good and the crystallinity of the MEB‐derived 2DLMs is high as compared to the CVD and PLD‐grown 2DLMs. As shown in Figure 4b, high‐resolution scanning transmission electron microscope image and the corresponding sharp selected area electron diffraction pattern distinctly demonstrate the highly ordered atomic arrangement of the MBE‐grown 2D (Bi_1−_ *_x_* Sb*_x_*)_2_Se_3_ alloy.^[^ ^172^ ^]^ This makes MBE a promising technique for the fundamental research on the exotic physics of 2DLM alloys. For example, the Fermi level of the above MBE‐grown (Bi_1−_ *_x_* Sb*_x_*)_2_Se_3_ topological insulator can be well tailored by precisely tuning the Sb content (Figure 4c), providing a powerful route to suppress the bulk transport signals. Furthermore, in contrast to conventional heteroepitaxy, the MBE growth of 2DLMs has the benefit of the relief of strict requirement of lattice matching constrains confirming to their dangling‐bond‐free surface. However, the time‐consuming vacuuming process for an ultrahigh vacuum growth environment, which brings about low growth efficiency, and exorbitant instrument cost are disadvantages hindering its widespread industrial application.

**Figure 5:**
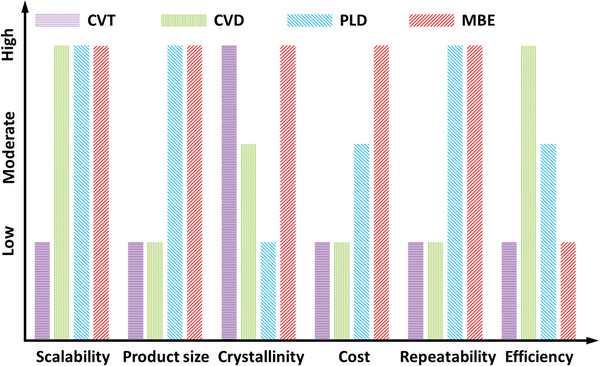
A schematic summary of the advantages and disadvantages of CVT, CVD, PLD, and MBE.

On the whole, CVT, CVD, PLD, and MBE have proven as promising techniques for preparing 2DLM alloys with unique advantages, despite that there are still predicaments to be addressed prior to their practical industrial application. For clarity, the strengths and shortcomings of these synthesis technologies, which are drawn from this section, have been laconically summarized in **Figure** **5**.

## 3 2D Layered Material Alloys for Electronic Device Applications

### 3.1 2D Layered Material Alloys for Field‐Effect Transistors

**Figure 6:**
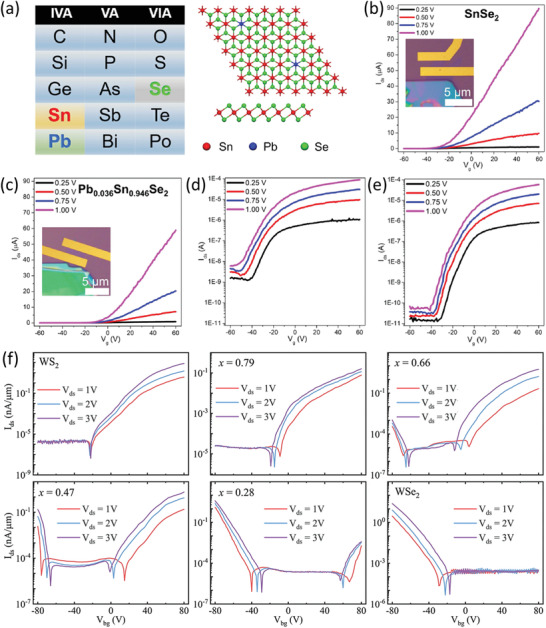
The application of 2DLM alloys for FETs. a) The periodic table of elements showing the positions of Sn, Pb, Se (left), and the schematic diagram of the crystal structure of Pb*_x_* Sn_1−_ *_x_* Se_2_ (right). b–e) Transfer curves of the monolayer SnSe_2_ and Pb*_x_* Sn_1−_ *_x_* Se_2_ FETs in linear and logarithmic scale. Source‐drain voltage: 0.25–1 V.^[^ ^122^ ^]^ a–e) Reproduced with permission.^[^ ^122^ ^]^ Copyright 2018, IOP Publishing Ltd. f) Transfer curves in logarithmic scale of a series of FETs based on monolayer WS_2_ *_x_* Se_2−2_ *_x_* alloys (*x* = 1, 0.79, 0.66, 0.47, 0.28, and 0, respectively). Source‐drain voltage: 1–3 V.^[^ ^180^ ^]^ Reproduced with permission.^[^ ^180^ ^]^ Copyright 2020, Royal Society of Chemistry.

FETs represent devices with the output channel current that can be controlled by the electrostatic gating.^[^ ^177^ ^]^ Basically, a gate voltage in vicinity to the transport channel gives rise to accumulation (depletion) of charge carriers, which results in the modulation of the channel conductance. Prior to further discussion, the important performance metrics of FETs are introduced. The on current and off current (also denoted as standby current) represent the drain current under the on and off states, respectively. The on/off ratio refers to the ratio of on current to off current. The field‐effect mobility (*μ*_EF_) represents the carrier mobility extracted from the transfer curve. It is calculated by

(1) $$\mu_{\mathrm{EF}}=\frac{Ld}{W\varepsilon_{0}\varepsilon_{r}V_{\mathrm{ds}}}\frac{dI_{\mathrm{ds}}}{dV_{g}}$$

where *L* and *W* are the length and width of the channel, *ε*_0_ is the vacuum dielectric constant, *ε*_r_ is the dielectric constant of the dielectric layer, *d* is the thickness of the dielectric layer, *V*_ds_ is the source‐drain voltage, *I*_ds_ is the source‐drain current, and *V*_g_ is the back gate voltage. Experimentally, 2DLMs inevitably suffer from crystal defects, which result in unintentional doping. This may degrade the properties of the corresponding 2DLM FETs. Fortunately, alloying is a promising route to tailor the charge carrier concentration by incorporating heterogeneous atoms with different valance state to those of the host 2DLM matrix. For example, in 2018, Liu et al. revealed that the device performances of a SnSe_2_ FET can be markedly improved by cation substitution with Pb atoms from the same periodic element group to Sn (**Figure** **6**a).^[^ ^122^ ^]^ As shown in Figure 6b,d, the pristine SnSe_2_ FET is difficult to be fully turned off by back gate coupling due to the high electron concentration, rendering a large standby current (≈10^−9^–10^−8^ A) related with low on/off ratio and high energy consumption. As shown in Figure 6c,e, the on/off ratio of a Pb_0.036_Sn_0.964_Se_2_ FET reaches ≈10^6^, which is two orders of magnitude higher than that of a pristine SnSe_2_ FET. Such dramatic improvement is ascribed to the alloying induced modulation of carrier concentration. Due to the lower valence state of Pb as compared to Sn, Pb atoms act as acceptors in the Pb*_x_* Sn_1−_ *_x_* Se_2_ alloy, partially compensating the excessively predominant electrons and thus leading to a decrease of electron concentration with respect to the intrinsically n‐type SnSe_2_. Specifically, electric transport measurements indicate that the substitution of ≈3.6% Sn atoms with Pb atoms results in a dramatic reduction of electron concentration by 6 times (from 2.08 × 10^12^ cm^−2^ to 3.31 × 10^11^ cm^−2^). This is also indirectly validated by a conspicuous positive shift of the threshold voltage. As a consequence, the standby current of the Pb_0.036_Sn_0.964_Se_2_ FET is markedly suppressed by two orders of magnitude (≈10^−11^–10^−10^ A) and the gate electric field induced switching character thus becomes much more pronounced. In particular, the field effect electron mobility of the SnSe_2_ and Pb_0.036_Sn_0.964_Se_2_ FETs is comparable (19.4 cm^2^ V^−1^ s^−1^ for the former and 15.7 cm^2^ V^−1^ s^−1^ for the latter). This is related to an intricate tradeoff between the impurity scattering and the electron‐electron scattering. That is, the incorporation of heterogeneous Pb atoms intensifies the impurity scattering, whereas the reduction of electron concentration suppresses the electron‐electron scattering, which conjointly result in roughly unchanged carrier mobility. Therefore, alloying is a powerful means to ameliorate the on/off ratio and energy consumption of 2DLM FETs whilst not compromising the on current (≈8.8 × 10^−5^ A for the SnSe_2_ FET and ≈5.9 × 10^−5^ A for the Pb_0.036_Sn_0.964_Se_2_ FET at a source‐drain voltage of 1 V).

On the other hand, alloying can also regulate the electric transport properties of 2DLMs via counteraction by deliberately coupling host materials with distinct charge‐carrier polarities. Typically, pristine S2DLMs have relatively steadfast electric transport properties. That is, normally only one type of charge polarity tends to be formed for a specific S2DLM. For example, MoS_2_ is a natively n‐type semiconductor, presumably because of the omnipresent electron‐donating sulfur vacancies,^[^ ^178^ ^]^ while WSe_2_ is a natively p‐type semiconductor.^[^ ^179^ ^]^ As the composition of 2DLM alloys can be markedly tuned over a wide range by virtue of the high miscibility of the host materials with a general stoichiometric formula and analogous crystal structure, it is plausible that the native propensity of electric transport characteristics of 2DLMs can be significantly modulated through alloy engineering.

In 2020, Sun et al. synthesized a series of uniform monolayer WS_2_ *_x_* Se_2−2_ *_x_* alloys with variable compositions through a salt‐assisted CVD method and systematically explored their electric transport characteristics.^[^ ^180^ ^]^ As shown in Figure 6f, as the Se content increases from 0% to 100%, the WS_2_ *_x_* Se_2−2_ *_x_* alloy gradually evolves from an n‐type semiconductor, and then to an ambipolar semiconductor, and finally to a p‐type semiconductor. The dramatic modulation of the electric transport property validates that alloying provides a flexible and facile route for the regulation of the charge‐carrier polarity of 2DLMs. In another study, a transition from n‐type conductance to p‐type conductance is achieved by incorporating Te atoms into MoS_2_.^[^ ^181^ ^]^ Inspiringly, a remarkable room‐temperature hole mobility of 182 cm^2^ V^−1^ s^−1^ is realized. Most recently, a bipolar to p‐type transition has been revealed in a V*_x_* W_1−_ *_x_* Se_2_ FET as the V content increases from 0% to 1.9%.^[^ ^182^ ^]^ In addition, this alloying strategy outperforms conventional approaches, such as, surface decoration,^[^ ^183^, ^184^, ^185^ ^]^ in terms of stability, as the heteroatoms in alloys are firmly secured by strong covalent bonding. This is of prodigious significance for the implementation of complicated CMOS circuits based on an individual material system, and it may substantially simplify the preparation of materials toward next‐generation microelectronic circuits.

### 3.2 2D Layered Material Alloys Based Diodes

Diodes refer to devices with unidirectional electric conduction capability, which is generally originated from the built‐in electric field of heterostructures.^[^ ^137^, ^186^, ^187^ ^]^ Basically, a high current flow is generated when the external applied voltage bears the opposite direction to the built‐in electric field, as it can significantly lower the interfacial potential barrier. On the contrary, the current flow is markedly suppressed when the external applied voltage bears the same direction to the built‐in electric field, as it can significantly elevate the interfacial potential barrier. An important performance metric of diodes is the rectification ratio (RR), which is defined as the ratio of forward current to reverse current. It can be expressed as

(2) $$RR_{\left|V\right|}=I_{V}/I_{-V}$$

*I*_V_ and *I*_−V_ are the current flow under forward voltage and reverse voltage, respectively. By virtue of the dangling‐bond‐free top/bottom surface, 2DLMs can be discretionarily stacked to fabricate various heterostructures without strict lattice matching constrains as those of conventional heterojunctions.^[^ ^188^, ^189^, ^190^, ^191^ ^]^ In addition, as discussed in the previous section, alloying can modulate the carrier concentration in a substantially broad range or even reverse the charge‐carrier polarity of semiconducting 2DLMs without compromising the layered crystal structures. For example, Liu et al. have demonstrated a p‐type to n‐type transition of the Nb*_x_* Re_1−_ *_x_* Se_2_ alloy as the Nb content increases from 0% to 12.5%.^[^ ^112^ ^]^ Therefore, alloying provides a viable option for the preparation of various building blocks toward constructing diodes.

**Figure 7:**
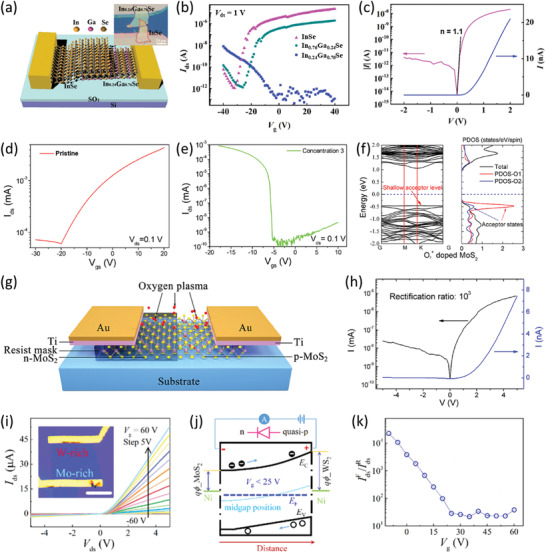
The application of 2DLM alloys for diodes. a) Schematic diagram of an InSe/In_0.24_Ga_0.76_Se diode. b) Transfer curves of In_1−_ *_x_* Ga*_x_* Se FETs with different contents of Ga (*x* = 0, 0.24, 0.76). c) *I*–*V* curves of an InSe/In_0.24_Ga_0.76_Se diode in logarithmic scale (pink) and linear scale (blue).^[^ ^192^ ^]^ a–c) Reproduced with permission.^[^ ^192^ ^]^ Copyright 2020, American Chemical Society. d,e) Transfer curves of the pristine and oxygen plasma treated MoS_2_ FETs. f) Band diagram and density of states of the O_2_^+^ doped MoS_2_. g) Schematic illustration of the fabrication of an in‐plane MoS_2_/MoS_2−_ *_x_* O*_x_* heterojunction. h) *I*–*V* curve of a MoS_2_/MoS_2−_ *_x_* O*_x_* diode in logarithmic scale (black) and linear scale (blue).^[^ ^198^ ^]^ d–h) Reproduced with permission.^[^ ^198^ ^]^ Copyright 2019, IOP Publishing Ltd. i) *I*–*V* curves of a compositionally graded Mo_1−_ *_x_* W*_x_* S_2_ diode under various gate voltages. The inset shows the optical microscope image of a typical device. j) Band diagram illustrating the working mechanism. k) Rectification ratio as a function of gate voltage.^[^ ^199^ ^]^ i–k) Reproduced with permission.^[^ ^199^ ^]^ Copyright 2019, American Chemical Society.

Most recently, Yu et al. have developed a high‐performance diode based on an InSe/In_0.24_Ga_0.76_Se heterojunction (**Figure** **7**a).^[^ ^192^ ^]^ Specifically, as the Ga content increases from 0% to 76%, the In_1−_ *_x_* Ga*_x_* Se alloy gradually evolves from an n‐type semiconductor to a p‐type semiconductor (Figure 7b), thus providing both p‐type and n‐type building blocks for constructing p‐n junctions. In particular, the fabricated InSe/In_0.24_Ga_0.76_Se diode achieves a strong current rectifying behavior with a rectification ratio of 3.6 × 10^3^ at a source‐drain voltage of ±1 V and an ideality factor of 1.1 (Figure 7c). These superior device performances can be associated with the high‐quality heterointerface enabled by the dangling‐bond‐free interface of the 2DLM building blocks. On the whole, this study presents a flexible and versatile paradigm for constructing high‐performance 2D diodes. In another study, Fan et al. realized tunable interfacial quantum tunneling based on a V*_x_* W_1−_ *_x_* Se_2_/SnSe_2_ diode.^[^ ^193^ ^]^ As the V content increases, the original type‐II band alignment gradually evolves into type‐III band alignment. This gives rise to a variety of diode behaviors such as forward diode, backward diode, negative differential resistance, and ohmic resistance, which is attractive for the demonstration of multifunctional devices. Inspiringly, aside from p(n)‐type to n(p)‐type transition, semiconductor‐to‐metal transition has also been achieved in a host of 2DLM alloys such as MoSe_2_ *_x_* Te_2(1−_ *_x_*_)_,^[^ ^113^ ^]^ WS_2(1−_ *_x_*_)_Te_2_ *_x_*,^[^ ^194^ ^]^ WSe_2(1−_ *_x_*_)_Te_2_ *_x_*,^[^ ^114^, ^195^ ^]^ and Mo_1−_ *_x_* Re*_x_* Se_2_,^[^ ^196^ ^]^ just to name a few. Therefore, these 2D alloys provide prospective material platforms for the construction of Schottky diodes in the future. On the other hand, this strategy is applicable to 2D/3D heterojunctions as well. For example, in a recent work by Chanhan et al., it is found that the rectification ratio of a Re*_x_* Sn_1−_ *_x_* Se_2_/p‐Si diode is ≈3 times higher than that of a pristine SnSe_2_/p‐Si diode.^[^ ^197^ ^]^ The ameliorated device property is ascribed to the alloying induced upshift of the Fermi level to conduction band, which renders elevated interfacial build‐in electric field.

In spite of the immense success of the above strategy, it can only produce diodes based on vertically stacked heterostructures, whereas in‐plane diodes are unavailable for this technique. To address this issue, in 2019, Wu et al. developed a selected area plasma doping approach for preparing high‐performance atomically thin p‐n junctions.^[^ ^198^ ^]^ Basically, pristine MoS_2_ is a typical n‐type semiconductor (Figure 7d). After doping with O_2_ plasma, the resulting MoS_2−_ *_x_* O*_x_* alloy becomes a p‐type semiconductor (Figure 7e). In addition, the room‐temperature field effect hole mobility of the MoS_2−_ *_x_* O*_x_* alloy is as high as 135 cm^2^ V^−1^ s^−1^. First‐principle calculations reveal that the incorporation of oxygen into the MoS_2_ host lattice leads to the generation of a shallow acceptor level slightly above the valance band maximum (VBM), which accounts for the p‐type character of MoS_2−_ *_x_* O*_x_* (Figure 7f). As shown in Figure 7g, by performing selected area plasma treatment on MoS_2_, an in‐plane MoS_2−_ *_x_* O*_x_*/MoS_2_ p‐n junction is naturally formed. Importantly, this device displays prominent rectification characteristic with a rectification ratio exceeding 10^3^ (Figure 7h).

Considering that heterogeneous composition distribution may result in anisotropic electronic structure, in‐plane diodes can also be implemented based on the compositionally graded 2DLM alloy channels. For example, in 2019, Yang et al. fabricated a diode by capitalizing on compositionally graded 2D Mo_1−_ *_x_* W*_x_* S_2_ alloy channel.^[^ ^199^ ^]^ As shown in Figure 7i, the Mo_1−_ *_x_* W*_x_* S_2_ device exhibits distinct rectification characteristic along the direction with gradually varied composition. This is originated from the shift of the Fermi level caused by the composition change (Figure 7j). Specifically, the conduction band minimum (CBM) and VBM of WS_2_ are both higher than those of MoS_2_. Therefore, as the W content increases, the CBM and VBM of Mo_1−_ *_x_* W*_x_* S_2_ gradually upshift. As a consequence, the Fermi level of Mo_1−_ *_x_* W*_x_* S_2_ gradually shifts to the VBM with increasing W content. Therefore, the conduction polarity of the Mo_1−_ *_x_* W*_x_* S_2_ channel will gradually evolve from n‐type dominant type at the Mo‐rich side to p‐type dominant type at the W‐rich side, thus forming a quasi‐p‐n junction and exhibiting distinct rectification characteristic. Beyond that, since the atomically thin 2DLMs have excellent electrostatic tunability, the rectification ratio of this device can be further modulated by leveraging the gate voltage. As shown in Figure 7h, as the gate voltage evolves from 60 to −10 V, the rectification ratio first remains roughly unchanged and then increases dramatically. Specifically, under a gate voltage of −10 V, its rectification ratio reaches a high value of 2.2 × 10^4^. As a whole, this study depicts a novel route circumventing the complicated fabrication process of p‐n junctions or Schottky junctions to realize the rectification effect, which opens new opportunities for the implementation of high‐performance 2D diodes.

### 3.3 2D Layered Material Alloys Based Logic Inverters

Logic inverters represent devices output a voltage signal with the opposite logic level to its input.^[^ ^200^ ^]^ The specific working principle of a logic inverter depends on its circuit. Basically, an inverter technology is based on a proportioned logic, where the output voltage depends on the voltage portion between two transistors connected in series. The key performance metric of logic inverters is the voltage gain, which is defined as the ratio of the output voltage to the input voltage. Logic inverters are essential functional units in the modern integrated digital and analog circuitries toward extensive applications such as artificial intelligence and the internet of everything.^[^ ^201^ ^]^ Generally, the implementation of high‐performance CMOS logic inverters requires both p‐type and n‐type semiconductor channels. In spite of the grand promise of 2DLMs for FETs, such as, intrinsic immunity to short‐channel effects and outstanding gate tunability, building complementary logic circuitry has proven to be challenging regarding the difficulty of realizing high‐performance n‐type and p‐type FETs by exploiting an individual kind of 2D semiconductor. Therefore, exploitation of multiple materials with various intrinsic charge‐carrier polarities^[^ ^202^, ^203^, ^204^ ^]^ and specific post‐synthesis treatments such as solution processing,^[^ ^205^ ^]^ laser irradiation,^[^ ^206^ ^]^ and doping^[^ ^207^ ^]^ have been developed to address this issue. For example, in 2017, Lee et al. fabricated a high‐performance inverter with a voltage gain of ≈40 through integrating the n‐type InGaZnO FET and p‐type MoTe_2_ FET.^[^ ^204^ ^]^ In another work, Wan et al. constructed an inverter by exploiting p‐type and n‐type WSe_2_ FETs produced by the surface charge transfer assisted doping.^[^ ^208^ ^]^ Specifically, n‐type doping is realized by using benzyl viologen as dopant, and p‐type doping is realized by exploiting ozone exposure. Importantly, the voltage gain of this device reaches ≈32 at a supply voltage of 5 V. Despite of the significant advancements, these strategies either entail complex material synthesis or intricate post‐synthesis material processing or suffer from poor reliability, making them inconducive to practical application.

**Figure 8:**
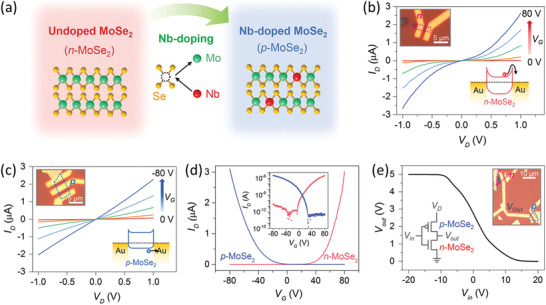
The application of 2DLM alloys for logic inverters. a) Schematic illustration of the alloying induced transition of charge‐carrier polarity. b,c) *I*–*V* curves of the MoSe_2_ and Nb*_x_* Mo_1−_ *_x_* Se_2_ FETs under various gate voltages from 0 to 80 V. d) Transfer curves of the MoSe_2_ and Nb*_x_* Mo_1−_ *_x_* Se_2_ FETs. The inset presents the data in logarithmic scale. e) Conversion characteristic of an inverter built of the n‐type MoSe_2_ and p‐type Nb*_x_* Mo_1−_ *_x_* Se_2_ FETs.^[^ ^209^ ^]^ Reproduced with permission.^[^ ^209^ ^]^ Copyright 2015, John Wiley and Sons, Inc.

Alternatively, alloying has proven as a powerful tool to modulate the charge‐carrier polarity of 2DLMs without significantly complicating the material preparation. In this point of view, it represents a flexible and economical protocol to implement complementary logic inverters. As a pioneering attempt, Jin et al. developed complementary inverters based on pristine TMDCs and the corresponding TMDC based alloys.^[^ ^209^ ^]^ As schematically shown in **Figure** **8**a, pristine MoSe_2_ is a typical n‐type semiconductor. In contrast, after the substitution of a portion of Mo atoms by Nb atoms, the resulting Nb*_x_* Mo_1−_ *_x_* Se_2_ alloy becomes a typical p‐type semiconductor. This conversion of charge‐carrier polarity is definitely validated by systematic electric measurements, where opposite trends in transfer curves are validated for the MoSe_2_ and Nb*_x_* Mo_1−_ *_x_* Se_2_ FETs (Figure 8b–d). Importantly, a COMS logic inverter, which demonstrates distinct on/off states, has been effectuated based on them (Figure 8e). Although the voltage gain of this inverter is unsatisfactory in the current stage, in theory, its performance can be significantly modulated by simply adjusting the alloy composition, and there is thus still huge room for further improvement. On the whole, these experimental findings establish that alloying opens a flexible avenue to realize both p‐type conductance and n‐type conductance in a homogeneous material channel. Therefore, it can markedly streamline the material production for the realization of the next‐generation CMOS logic inverters and it is worthy of in‐depth exploration in the future.

## 4 2D Layered Material Alloys for Photodetection

Photodetectors are devices converting optical signals into electric signals, which are critical function units of extensive applications spanning LiFi communication,^[^ ^210^ ^]^ target motion tracking,^[^ ^211^ ^]^ optical imaging,^[^ ^212^ ^]^ optoelectronic memory,^[^ ^213^ ^]^ biometric authentication,^[^ ^214^ ^]^ intelligent robots,^[^ ^215^ ^]^ internet of things,^[^ ^95^ ^]^ just to name a few. Basically, photocarriers are generated upon light excitation with sufficiently high photon energy, where electrons transit from conduction band to valence band of the photoactive materials. These excess free carriers can modulate the overall electric conductance of the photoactive channel and thus give rise to net photocurrent across the circuit. Prior to the further discussion, the important performance metrics of photodetectors are introduced. Responsivity (*R*) refers to the generated photocurrent upon illumination with per unit of energy. It is calculated by

(3) $$R=\frac{I_{\mathrm{Light}}-I_{\mathrm{Dark}}}{PA}$$

where *I*_Light_ and *I*_Dark_ are the channel current under illumination and in dark, *P* is incident light's power density, and *A* is the effective photosensitive area. External quantum efficiency (EQE) reflects the efficiency of incident photons to generate effective photocarriers. It is equal to the number of collected charge carriers by external circuits upon excitation of a single incident photon. It is calculated by

(4) $$\mathrm{EQE}\left(\lambda \right)=\frac{\mathrm{hcR}}{\lambda e}$$

where *h* is the Planck constant, *c* is the velocity of light, *λ* is incident light's wavelength, and *e* is the elementary charge. Detectivity (D*) is employed to evaluate a PD's capability to distinguish the weak light signal. It is calculated by

(5) $$D^{*}=\frac{\sqrt{A}R}{\sqrt{2eI_{\mathrm{Dark}}}}$$

Since both the optical and electric properties of 2DLMs can be markedly modulated by adjusting their composition, alloy engineering represents a powerful route to tailor the performance of 2DLM photodetectors.

### 4.1 Suppression of Deep‐Level Defect States through Alloy Engineering

Generally, 2DLMs, especially those synthesized under high reaction temperatures or those having been deposited in ambient environment for a substantially long period, will inevitably suffer from numerous crystal defects induced by thermal damage and air aging. Among various defects, the anion vacancies are the most likely to form regarding the lowest formation energy.^[^ ^216^, ^217^, ^218^ ^]^ For example, Qiu et al. have identified the existence of numerous S vacancies (≈3.6 × 10^13^ cm^−2^) even in the mechanically exfoliated monocrystalline 2D MoS_2_ nanosheets by exploiting the aberration‐corrected transmission electron microscopy measurements.^[^ ^219^ ^]^ In addition, these vacancies may accelerate the oxidation of 2DLMs due to the unsaturated bond induced high chemical activity,^[^ ^218^ ^]^ which introduces a myriad of deep‐level defect states inside the bandgap. These mid‐gap states are scattering centers for free carriers and high‐efficiency recombination centers for photoexcited electron–hole pairs. They are detrimental to the electric and optical properties of 2DLMs. Furthermore, the large surface‐to‐volume ratio makes 2DLMs much more susceptible to these defects as compared to the bulk counterparts. Theory investigation has indicated that the defect state assisted annihilation of exciton in 2D TMDCs is 100–1000 times faster than that without defects.^[^ ^220^ ^]^ Most recently, first‐principles calculations have predicted a dramatic degradation of carrier mobility by two orders of magnitude as the vacancy concentration of 2D MoS_2_ increases from 0.1% to 3%.^[^ ^221^ ^]^ Both of these factors are inconducive to the accumulation and transport of photocarriers.

A series of post‐synthesis approaches, such as adsorption of sulfur adatom clusters,^[^ ^222^, ^223^ ^]^ laser irradiation,^[^ ^224^ ^]^ and superacid treatment,^[^ ^225^ ^]^ have by far been developed for defect passivation. However, these treatments are complicated, and the efficacies are limited. In addition, the reliability of these approaches remains another severe issue impeding their practical application. For example, the healing of S vacancies of MoS_2_ by oxygen molecules can be easily invalidated by either vacuum or light excitation.^[^ ^224^ ^]^ Furthermore, it is unrealistic to completely eliminate all the crystal defects even with thorough‐paced post‐processing.

**Figure 9:**
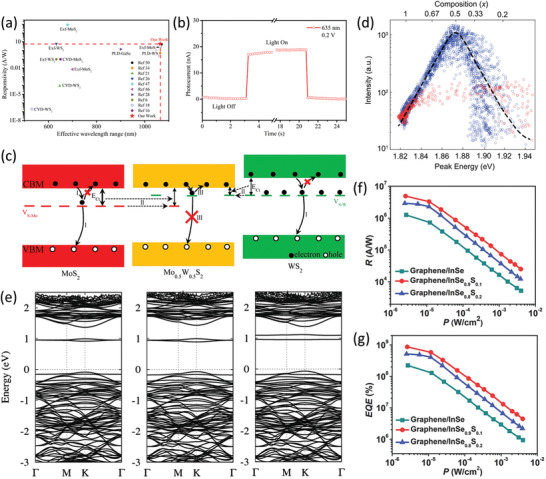
Suppression of deep‐level defect states by 2DLM alloy for improved photosensitivity. a) A summary of the responsivity of a PLD‐produced Mo_0.5_W_0.5_S_2_ alloy photodetector and other photodetectors based on binary Mo‐/W‐based dichalcogenides produced by various methods. b) Temporal photoresponse of a PLD‐produced Mo_0.5_W_0.5_S_2_ photodetector. c) Band diagram illustrating the working mechanism of the suppression of deep‐level defect states by alloy engineering.^[^ ^155^ ^]^ a–c) Reproduced with permission.^[^ ^155^ ^]^ Copyright 2016, American Chemical Society. d) PL intensity of the Mo*_x_* W_1−_ *_x_* S_2_ alloy as a function of Mo composition.^[^ ^230^ ^]^ Reproduced with permission.^[^ ^230^ ^]^ Copyright 2018, Nature Publishing Group. e) Band diagrams showing the defect levels of Se vacancy in MoSe_2_ (left) and Se vacancy in Mo_0.75_W_0.25_Se_2_ surrounded by Mo atoms (middle) or W atoms (right).^[^ ^231^ ^]^ Reproduced with permission.^[^ ^231^ ^]^ Copyright 2017, John Wiley and Sons, Inc. f,g) Responsivity and EQE as a function of power density of graphene/InSe_1−_ *_x_* S*_x_* (*x* = 0.1, 0.2, 0.3) heterojunction photodetectors.^[^ ^240^ ^]^ f,g) Reproduced with permission.^[^ ^240^ ^]^ Copyright 2020, Royal Society of Chemistry.

Fortunately, alloying sheds light by leveraging energy band engineering. Through the rational design of alloys, the deep‐level defect states of 2D semiconductors can be significantly tailored by tuning their relative energy levels in the bandgap. To this end, in 2016, Yao et al. synthesized centimeter‐scale 2D Mo_0.5_W_0.5_S_2_ alloy via PLD and systematically investigated its photoresponse properties.^[^ ^155^ ^]^ Inspiringly, the 2D Mo_0.5_W_0.5_S_2_ alloy showcases a high Hall electron mobility of 35 cm^2^ V^−1^ s^−1^, which is ≈2 orders of magnitude higher than that of the reported PLD‐grown pristine WS_2_^[^ ^226^ ^]^ and it is even 2 times higher than that of the annealed PLD‐grown WS_2_.^[^ ^227^ ^]^ As a consequence, the Mo_0.5_W_0.5_S_2_ photodetector achieves a high responsivity of 5.8 A W^−1^ as well as, an excellent EQE of 1135%, and the response/recovery time (*τ*_rise_/*τ*_decay_) is less than 150 ms/150 ms (**Figure** **9**a,b). These performance metrics are much superior to those of the PLD‐derived 2D WS_2_ photodetectors (0.51 A W^−1^, 101%, 4.1 s/4.9 s)^[^ ^163^ ^]^ and the CVD‐derived single domain ones (3.2 mA W^−1^, 0.16 s/0.17 s).^[^ ^228^ ^]^ The ameliorated device performances are mainly ascribed to the suppression of deep‐level defect states through alloy engineering, as is explained in detail below (Figure 9c). According to previous reports,^[^ ^109^, ^229^ ^]^ MoS_2_ and WS_2_ form a type‐II band alignment. In addition, the CBM of Mo_0.5_W_0.5_S_2_ is close to that of MoS_2_, while it is much lower as compared to that of WS_2_.^[^ ^109^ ^]^ In addition, the corresponding energy levels of the defect states of Mo_0.5_W_0.5_S_2_ are roughly unchanged as compared to those of WS_2_ and MoS_2_, since these states are largely dominated by the nearest‐neighbor atoms. Therefore, the electron ionization energy (i.e., the energy difference between CBM and defect level) of the defect states V_S–W_ (S vacancy in vicinity to W atoms) of Mo_0.5_W_0.5_S_2_ is much lower than those of WS_2_, while the electron ionization energy of defect states V_S–Mo_ (S vacancy in vicinity to Mo atoms) of Mo_0.5_W_0.5_S_2_ is close to those of MoS_2_. It is to be emphasized that the former kind of defect is dominant in the Mo_0.5_W_0.5_S_2_ alloy due to its relatively low formation energy. That is, most defects in Mo_0.5_W_0.5_S_2_ are shallow level defect states. As a consequence, the defect induced scattering and recombination of photocarriers are markedly suppressed, which thus gives rise to improved response rate and photosensitivity. The suppression of deep‐level defect states has also been validated by the increased quantum yield of PL of Mo_0.5_W_0.5_S_2_ as compared to those of pristine MoS_2_ and WS_2_ in another independent study by Bogaert and his co‐authors (Figure 9d).^[^ ^230^ ^]^ In a subsequent study, Li et al. revealed that both the number of defects and defect levels can be suppressed in the 2D TMDC alloys.^[^ ^231^ ^]^ Specifically, the Se vacancy concentration in the CVD‐grown Mo_1−_ *_x_* W*_x_* Se_2_ is suppressed by 50% as compared to that of MoSe_2_. In addition, the PL intensity and carrier lifetime of Mo_1−_ *_x_* W*_x_* Se_2_ are increased by 10 and 3 times, respectively. Density function theory calculations reveal that the incorporation of W into the MoSe_2_ skeleton can raise the energy levels of deep‐level defect states and shift them to the CBM, thus effectuating reduced electron ionization energy (Figure 9e). These paradigms demonstrate the grand potential of the alloying approach for regulating the photosensitivity of 2DLMs by tailoring the defect states. Following these advancements, Liu et al. demonstrated a high field‐effect electron mobility of 30 cm^2^ V^−1^ s^−1^ in the CVD‐grown monolayer Mo_0.5_W_0.5_S_2_ alloy.^[^ ^232^ ^]^ In addition, Lim et al. achieved markedly boosted photoresponse in a MoS_1.15_Se_0.85_ photodetector as compared to that of the pristine MoS_2_ and MoSe_2_ photodetectors.^[^ ^233^ ^]^ Specifically, the photocurrent of the former is ≈1 order of magnitude higher than that of the latter. Under a source‐drain voltage of 10 V, the responsivity of the MoS_1.15_Se_0.85_ photodetector reaches 2.06 A W^−1^, which is higher than those of commercial Si and Ge photodetectors (≈0.1 A W^−1^). In another work, Mo et al. found that the response time of a Mo_1−_ *_x_* Sn*_x_* S_2_ photodetector can be markedly shortened with increasing Sn content.^[^ ^234^ ^]^ Specifically, the response/recovery times of a Mo_0.74_Sn_0.26_S_2_ photodetector (20/23 ms) are 2–3 orders of magnitude shorter than those of a pristine MoS_2_ photodetector (2.12/0.62 s). Most recently, Kang et al. have demonstrated that the photoresponse of ReS_2_ *_x_* Se_2(1−_ *_x_*_)_ alloy is also markedly modulated as compared to that of pristine ReS_2_ and ReSe_2_.^[^ ^145^ ^]^ Under 532 nm illumination, the optimized responsivity of a ReS_0.92_Se_1.08_ photodetector reaches 0.25 A W^−1^, which is several orders of magnitude higher than that of a pristine ReS_2_ photodetector (3 mA W^−1^), and it is also slightly higher than that of a pristine ReSe_2_ photodetector (0.14 A W^−1^). In addition, as the content of Se increases, the response time first decreases and then slightly increases, whereas the recovery time first decreases and then remains roughly unchanged. Specifically, at a Se content of 54% (i.e., ReS_0.92_Se_1.08_), the device simultaneously achieves the shortest response time and recovery time (15 m/15 ms), which are superior to that of pristine ReS_2_ (60 ms/350 ms) and ReS_2_ (35 ms/34 ms) photodetectors. Thus far, ameliorated device performances have been extensively demonstrated in numerous 2DLM alloys such as In*_x_* Sn_1−_ *_x_* S_2_,^[^ ^120^ ^]^ SnS_2−_ *_x_* Se*_x_*,^[^ ^235^ ^]^ and MoS_2(1−_ *_x_*_)_Se_2_ *_x_*,^[^ ^236^ ^]^ revealing broad expandability of this approach.

In a recent work, Gao et al. further developed the alloying methodology for 2D/3D heterojunction photodetectors.^[^ ^237^ ^]^ By virtue of the suppression of deep‐level defect states, 2D SnS_0.27_Se_0.75_ exhibits the highest carrier mobility among the SnS_1−_ *_x_* Se*_x_* alloys with different S content from 0% to 100%, making it the most compelling candidate for 2D/3D heterojunction photodetectors. Under a self‐powered working mode, the fabricated SnS_0.25_Se_0.75_/Si photodetector achieves high responsivity, detectivity and on/off ratio of 377 mA W^−1^, 10^11^ Jones (1 Jones = 1 cm Hz^1/2^ W^−1^) and 450, respectively. In addition, the response/recovery times are 4.7/3.9 ms, which are several orders of magnitude shorter than those of pristine SnS^[^ ^238^ ^]^ and SnSe^[^ ^239^ ^]^ photodetectors. In addition, alloying can also be applied to the heterojunction based MSM photodetectors. Most recently, Hao et al. have fabricated a series of graphene/InSe_1−_ *_x_* S*_x_* photodetectors (*x* = 0, 0.1, 0.2) and systematically investigated their photoresponse.^[^ ^240^ ^]^ Taking advantages of the strong light–matter interactions of InSe_1−_ *_x_* S*_x_* and high‐efficiency carrier transport of graphene, these devices demonstrate remarkable photosensitivity. In particular, the graphene/InSe_0.9_S_0.1_ photodetector achieves an outstanding responsivity of ≈4.9 × 10^6^ A W^−1^ and a remarkable EQE of 8.7 × 10^8^%, which are much higher than those of the graphene/InSe (≈1.2 × 10^6^ A W^−1^, 2.1 × 10^8^%) and graphene/InSe_0.8_S_0.2_ (≈2.9 × 10^6^ A W^−1^, 5.1 × 10^8^%) photodetectors (Figure 9f,g). However, the working principle for such amelioration has not been clearly unveiled. It can be originated from a synergy effect of the modulated defect states and band alignment between graphene and InSe_1−_ *_x_* S*_x_*, which deserves in‐depth exploration in the future.

### 4.2 Broadening of the Effective Wavelength Range through Alloy Engineering

Broadband photodetectors are of pivotal importance for improving the versatility and integration level of modern optoelectronic systems by virtue of their broad applicability for a wide breadth of working environment.^[^ ^238^, ^239^, ^241^, ^242^, ^243^ ^]^ Theoretically, the effective wavelength range for photodetection of a semiconductor is determined by its bandgap size (*E*_g_, the unit is electron volt), where only incident light with photon energy larger than the bandgap of semiconductor can excite electron–hole pairs. Therefore, the cutoff wavelength (*λ*) is generally dictated by

(6) $$\lambda \left(nm\right)=1243/E_{g}$$

Since the extensively explored 2D TMDCs and post‐transition metal chalcogenides normally possess sizable bandgaps in the range of 1–2 eV, the effective wavelength range of the corresponding photodetectors is largely limited to the visible to near‐infrared range.^[^ ^217^, ^244^, ^245^ ^]^ In recent years, a variety of strategies such as strain engineering,^[^ ^246^, ^247^ ^]^ defect engineering,^[^ ^248^ ^]^ coupling of ferroelectric dielectric layers with 2DLMs,^[^ ^249^ ^]^ and heterogeneous systems^[^ ^250^, ^251^ ^]^ have been developed to broaden the effective wavelength range of 2DLM photodetectors. However, these strategies are either complicated in processing or at the compromise of other device performances such as responsivity, response rate and on/off ratio. For example, Xie et al. recently reported the success of room‐temperature detection of 2.52 THz light illumination based on a Mo‐vacancy‐rich 2D MoS_2.19_ photosensitive channel.^[^ ^248^ ^]^ However, this device's responsivity, response/recovery time and on/off ratio are only 10 mA W^−1^, 5.12 s/6.33 s and ≈1/100, which are far inferior to those of the reported defect‐free MoS_2_ photodetectors.^[^ ^252^ ^]^

**Figure 10:**
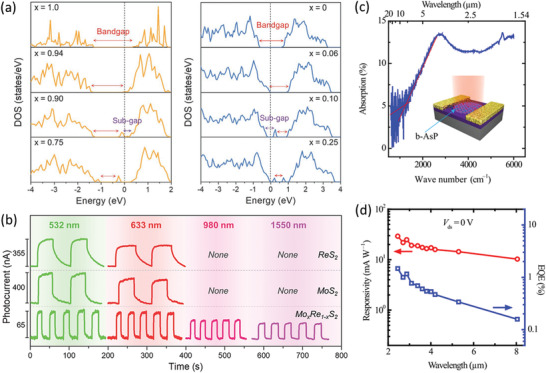
2DLM alloys for broadband photodetectors. a) Total density of states of the Mo*_x_* Re_1−_ *_x_* S_2_ alloys with different compositions. b) Photoswitching curves of the ReS_2_, MoS_2_, and Mo*_x_* Re_1−_ *_x_* S_2_ photodetectors under 532, 633, 980, and 1550 nm illumination.^[^ ^253^ ^]^ a,b) Reproduced with permission.^[^ ^253^ ^]^ Copyright 2020, John Wiley and Sons, Inc. c) Absorption spectrum of b‐As_0.83_P_0.17_. The inset presents a schematic diagram of a b‐As_0.83_P_0.17_ phototransistor. d) Responsivity (red) and EQE (blue) of a b‐As_0.83_P_0.17_ photodetector as a function of wavelength.^[^ ^259^ ^]^ c,d) Reproduced with permission.^[^ ^259^ ^]^ Copyright 2017, American Association for the Advancement of Science.

Incorporation of heterogeneous atoms into the pristine 2DLM matrix is a powerful route to introduce mid‐gap states. These intermediate states can participate in the excitation of carriers, which can thus reduce the lowest photon energy required for triggering the transition of electrons. As a result, these mid‐gap states can extend the effective wavelength range of 2DLMs without compromising the electric transport properties, provided little lattice defects, such as strain and distortion, are introduced. Therefore, in theory, alloy engineering represents a potential scheme to implement high‐performance broadband photodetection. In 2020, Deng et al. demonstrated broadband photodetection from 532 to 1550 nm based on a 2D Mo*_x_* Re_1−_ *_x_* S_2_ alloy photodetector.^[^ ^253^ ^]^ As shown in **Figure** **10**a, density functional theory calculations indicate that a mid‐gap band generates in the Mo*_x_* Re_1−_ *_x_* S_2_ alloy as *x* is below 0.9. As shown in Figure 10b, photoelectric measurements reveal that pristine ReS_2_ and MoS_2_ photodetectors exhibit negligible photoresponse to the 980 nm and 1550 nm illumination with photon energy below their bandgaps. In contrast, distinct photoswitching is demonstrated in a Mo*_x_* Re_1−_ *_x_* S_2_ photodetector upon both 980 and 1550 nm periodic illumination. It is worth emphasizing that in contrast to the defect engineered broadband 2DLM photodetectors with sluggish response rate,^[^ ^254^ ^]^ the response time of this Mo*_x_* Re_1−_ *_x_* S_2_ alloy device suffers no degradation as compared to those of the pristine ReS_2_ and MoS_2_ photodetectors. Similarly, Ye et al. have revealed the bandgap narrowing phenomenon of HfS_2_ by Te substitution.^[^ ^117^ ^]^ Specifically, the bandgap of HfS_2(1−_ *_x_*_)_Te_2_ *_x_* alloy decreases prominently from 1.7 to 0.88 eV as the Te content increases from 0 to 0.095. Consequently, the absorption spectrum of HfS_1.81_Te_0.19_ is markedly broadened from visible (730 nm) of binary HfS_2_ to short‐wavelength infrared (≈1400 nm), which covers the free‐space laser communications wavelength and the second near‐infrared region in medicine. Importantly, the corresponding HfS_1.81_Te_0.19_ photodetector demonstrates broadband photoresponse from 520 to 1310 nm, far beyond the limitation of pristine HfS_2_ photodetectors (<1000 nm).^[^ ^255^, ^256^, ^257^, ^258^ ^]^ In addition, this device also achieves a high responsivity of 2 A W^−1^. This value is significantly higher than many of the previously reported pristine HfS_2_ photodetectors (10^−2^–26.5 mA W^−1^).^[^ ^255^, ^256^, ^257^, ^258^ ^]^ Furthermore, a satisfactory response/recovery time of 8.8 ms/75 ms is demonstrated. These values are in the range of the reported results (*τ*_rise_ ≈ 130 µs–55 ms/*τ*_decay_ ≈ 155 µs/230 ms), and they can be further improved by strategies such as substrate passivation which is conducive to suppressing the interfacial defects.^[^ ^256^ ^]^

In addition to TMDCs, alloy engineering has been applied to the mono‐elemental 2DLMs toward broadband photodetection as well. For example, Long et al. have achieved room‐temperature broadband photodetectors by exploiting black arisen phosphorus alloy (b‐As_0.83_P_0.17_) as the photosensitive channels.^[^ ^259^ ^]^ As shown in Figure 10c, b‐As_0.83_P_0.17_ showcases broadband light absorption from near‐infrared to mid‐infrared with the absorption edge at ≈8.27 µm. Inspiringly, such wide light absorption range is far beyond the limit of both intrinsic black phosphorus^[^ ^260^ ^]^ and intrinsic black arisen,^[^ ^261^ ^]^ which is ascribed to the alloying induced bandgap modulation. As a consequence, the b‐As_0.83_P_0.17_ photodetector demonstrates room‐temperature broadband photoresponse from 405 nm to 8.05 µm, which is far beyond the limitation of pristine black phosphorus photodetectors (*λ* < 4.14 µm).^[^ ^262^, ^263^ ^]^ Both responsivity and EQE decrease monotonically with increasing wavelength of incident light in the infrared range (Figure 10d). Under a source‐drain voltage of 0 V, the responsivity lies in the range of 15–30 mA W^−1^, and the corresponding EQE is in the range of 0.1–2%. In addition, the b‐As_0.83_P_0.17_ photodetector achieves short response/recovery time of 0.54 ms/0.52 ms. The responsivity and EQE are comparable to those of the reported black phosphorus based self‐powered photodetectors, such as, Al‐doped b‐P (6.2 mA W^−1^)^[^ ^264^ ^]^ and gate‐defined b‐P (0.35 mA W^−1^).^[^ ^265^ ^]^ However, they still far lag behind those of commercial devices. Therefore, prior to practical application, strategies need to be developed to further improve the device performance.

On the whole, these accomplishments have conjointly established that alloying is a tangible strategy to broaden the effective wavelength range of 2DLMs based photodetectors without compromising other performance metrics, manifesting grand prospect for future multifunctional optoelectronic applications. Noteworthily, the alloying strategy is logically robust as the heteroatoms in alloys are firmly secured by strong covalent bonding. This is a prominent superiority as compared to the widely explored vacancy engineering strategy,^[^ ^254^, ^266^ ^]^ which will be invalidated easily by local structural rearrangements.

### 4.3 2D Layered Material Alloys toward Self‐Powered Photodetection

Self‐powered photodetectors bear the advantages of low standby energy dissipation, high on/off ratio, excellent detectivity, and fast response rate,^[^ ^267^, ^268^, ^269^, ^270^ ^]^ which endow them with the reliable availability to capture weak light signals and operate under a wide breadth of harsh working environment such as the space and abysmal sea. However, high‐quality heterostructures such as Schottky junctions and p‐n junctions are requisite to effectively separate the photogenerated electron–hole pairs. This requires the preparation of different components with widely diverse electric properties, which may make the material production complicated. For example, the construction of a TiO_2_/Spiro‐OMeTAD self‐powered photodetector requires a spray pyrolysis process to deposit TiO_2_ and a spin coating process to deposit Spiro‐OMeTAD.^[^ ^271^ ^]^ As is demonstrated above, alloying can significantly modulate the electric properties of 2D semiconductors and even give rise to the inversion of charge‐carrier polarity or semiconductor–metal (metal–semiconductor) phase transition.^[^ ^194^, ^272^, ^273^ ^]^ For example, as the S content decreases from 100% to 0%, the WS_2_ *_x_* Se_2−2_ *_x_* alloy will gradually evolve from a highly doped p‐type semiconductor, then to a lightly doped p‐type semiconductor, then to a lightly doped n‐type semiconductor, and finally to a highly doped n‐type semiconductor.^[^ ^272^ ^]^ In particular, the synthesis of 2DLM alloys with different atomic molar ratios can be commonly effectuated with an individual growth technique by simply modulating the growth conditions such as the substrate temperature, precursor temperature, mass ratio of precursors, as well as, positions of precursors and substrates, etc.^[^ ^141^, ^274^, ^275^ ^]^ Furthermore, the heterojunctions of 2DLMs can effectively suppress the generation‐recombination noise by virtue of the dangling‐bond‐free heterointerface. Therefore, alloy engineering provides a fascinating and flexible avenue for the preparation of high‐performance 2DLM based self‐powered heterojunction photodetectors.

**Figure 11:**
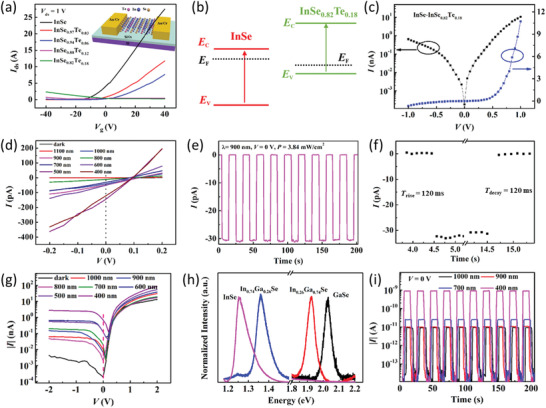
2DLM alloys for self‐powered photodetectors. a) Transfer curves of a series of InSe_1−_ *_x_* Te*_x_* FETs with different contents of Te. The inset presents the schematic diagram of an InSe_1−_ *_x_* Te*_x_* FET. b) Schematic illustration of the band alignment of an InSe/InSe_0.82_Te_0.18_ heterojunction. *E*_C_, *E*_V_, and *E*_F_ represent CBM, VBM, and Fermi level, respectively. c) *I*–*V* curves of an InSe/InSe_0.82_Te_0.18_ diode in dark in logarithmic scale (black) and in linear scale (blue). d) *I*–*V* curves of an InSe/InSe_0.82_Te_0.18_ photodetector in dark and under light illumination with wavelengths from 400 to 1100 nm. e) Photoswitching curve under periodic 900 nm light illumination at a self‐powered working mode. f) Temporal photoresponse.^[^ ^276^ ^]^ a–f) Reproduced with permission.^[^ ^276^ ^]^ Copyright 2020, Elsevier Ltd. g) *I*–*V* curves of an InSe/In_0.24_Ga_0.76_Se heterojunction photodetector in dark and under illumination with wavelengths from 400 to 1000 nm. h) PL spectra of In_1−_ *_x_* Ga*_x_* Se with different contents of Ga. i) Photoswtiching curves of an InSe/In_0.24_Ga_0.76_Se heterojunction photodetector under periodic light illumination with different wavelengths from 400 to 1000 nm.^[^ ^192^ ^]^ g–i) Reproduced with permission.^[^ ^192^ ^]^ Copyright 2020, American Chemical Society.

To this end, in 2020, Yu et al. developed a dry transfer method to fabricate a 2D p‐n diode based on an InSe/InSe_0.82_Te_0.18_ heterojunction and systematically evaluated its potency for self‐powered photodetection.^[^ ^276^ ^]^ As shown in **Figure** **11**a, the pristine InSe is an intrinsically n‐type semiconductor. Inspiringly, the electric transport property of the InSe_1−_ *_x_* Te*_x_* alloy is strongly dependent on the content of Te. As the Te content increases from 0% to 18%, the InSe_1−_ *_x_* Te*_x_* alloy gradually evolves from an n‐type semiconductor (e.g., InSe) to a p‐type semiconductor (e.g., InSe_0.82_Te_0.18_). It is noteworthy that the InSe_1−_ *_x_* Te*_x_* alloy with variable compositions can be produced by an individual growth technique, which significantly simplifies the device construction. In addition, in this composition range (0 ≤ *x* ≤ 0.18), the layered crystal structure of the InSe_1−_ *_x_* Te*_x_* alloy is well maintained. Therefore, the stack of 2D InSe and InSe_0.82_Te_0.18_ flacks results in the formation of a high‐quality dangling‐bond‐free p‐n junction (Figure 11b). As shown in Figure 11c, the InSe/InSe_0.82_Te_0.18_ heterojunction displays rectification characteristic with a rectification ratio of 10. Conforming to the dissociation of photogenerated electron–hole pairs by the built‐in electric field, the InSe/InSe_0.82_Te_0.18_ heterojunction can be exploited for self‐powered photodetection. As shown in Figure 11d, the InSe/InSe_0.82_Te_0.18_ heterojunction photodetector manifests pronounced photovoltaic characteristic under the light irradiation with different wavelengths in the range of 400–1000 nm. Specifically, upon 900 nm illumination, a responsivity of 14.1 mA W^−1^ is achieved, which, on the whole, lags behind the previously reported InSe‐based self‐powered heterojunction photodetectors (13.8–110 mA W^−1^).^[^ ^277^, ^278^, ^279^, ^280^ ^]^ The moderate performance is probably associated with the low interfacial built‐in electric field due to the far from optimized band alignment, which has also been validated by the inapparent rectification characteristic. In addition, this device not only displays a stable and reproducible photoresponse (Figure 11e), but also showcases short response/recovery time less than 120 ms/120 ms (Figure 11f). Following this success, the same research group fabricated an InSe/In_0.24_Ga_0.76_Se self‐powered photodetector.^[^ ^192^ ^]^ In contrast to the incipient attempts focusing on alloying of chalcogen elements, this study dedicates to the alloying of metal elements. Similarly, as the Ga content increases from 0% to 76%, the In_1−_ *_x_* Ga*_x_* Se alloy gradually transforms from an n‐type semiconductor to a p‐type semiconductor. As a result, the InSe/In_0.24_Ga_0.76_Se diode without gate modulation achieves a large rectification ratio in excess of 10^3^ (Figure 11g). In addition, it exhibits pronounced photoresponse to illumination with wavelengths from 400 to 1000 nm, which is probably associated with the complementary optical properties of InSe and In_0.24_Ga_0.76_Se (Figure 11h). Under a self‐powered working mode, this device demonstrates pronounced photoswitching behavior with a remarkable on/off ratio of ≈10–10^5^ (Figure 11i). In addition, its optimal responsivity reaches 0.12 A W^−1^. It is highly competitive among the state‐of‐the‐art 2D InSe based self‐powered heterojunction photodetectors, such as, GaSe/InSe (21 mA W^−1^),^[^ ^277^ ^]^ GaTe/InSe (13.8 mA W^−1^),^[^ ^279^ ^]^ InSe/MoS_2_,^[^ ^280^ ^]^ and Se/InSe (110 mA W^−1^).^[^ ^278^ ^]^ Importantly, the corresponding detectivity reaches 1.55 × 10^12^ Jones, which is also comparable to those of the above InSe based devices (1.08 × 10^10^−2.2 × 10^12^ Jones).

On the whole, the success of these studies has undoubtedly established that 2DLM alloys based heterostructures can provide abundant platforms for constructing high‐performance self‐powered photodetectors. In spite of the somewhat mediocre device performances in the current stage, which are probably ascribed to the far from optimized energy band structures, these pioneering studies present a novel, facile, and flexible gateway for the realization of self‐powered photodetectors through alloy engineering. In the future, the band alignments of the 2DLM alloys based heterostructures, including the CBM offset, VBM offset, and Fermi level offset, can be further optimized by simply tuning the components of the alloys, which promises a broad degree of freedom for the implementation of advanced self‐driven photoelectric devices.

### 4.4 Compositionally Graded 2D Layered Material Alloys toward Improved Photosensitivity

As for the photoconductive type photodetectors, the photoresponse is proportional to the lifetime of the photocarriers, which is dictated by

(7) $$I_{P}\propto \Delta n\mu \tau$$

where *I*_P_ is the photocurrent, Δ*n* is the concentration of photocarriers, *μ* is the carrier mobility, and *τ* is the carrier lifetime.^[^ ^281^ ^]^ Thus far, it has been extensively demonstrated that coupling semiconductor quantum dots with 2DLM channels can markedly prolong the lifetime of photocarriers by spatially separating the photogenerated electron–hole pairs, which results in remarkably high photogain.^[^ ^282^ ^]^ However, this approach suffers fatal challenges, such as slow response rate due to the sluggish detrapping process of photocarriers seized by the quantum dots. By sweeping the photogenerated electrons and holes in opposite directions, the built‐in electric field of heterojunctions and homojunctions, including Schottky junctions and p‐n junctions, can contribute to high‐efficiency separation of photogenerated electron–hole pairs as well.^[^ ^283^ ^]^ Nevertheless, it also has its own shortcomings. For example, only the photocarriers in vicinity of the heterointerface can be effectively swept and separated. A large amount of photocarriers will recombine before being extracted to the external circuit, especially for the in‐plane heterojunction and homojunction photodetectors.^[^ ^284^, ^285^ ^]^ This leads to relatively low responsivity. For example, the responsivity of a lateral MoS_2_/WS_2_ photodetector is as low as 4.36 mA W^−1^.^[^ ^284^ ^]^ To address this issue, Lu et al. introduced periodic built‐in electric field across the whole 2D photosensitive channel by deliberately coupling a 2D In_2_S_3_ flake with a SiO_2_ nanograting array, where strain generates in the unsupported In_2_S_3_.^[^ ^286^ ^]^ As a result, the In_2_S_3_/SiO_2_ photodetector achieves an excellent responsivity of 1810 A W^−1^ and a remarkable detectivity of 2.09 × 10^15^ Jones. Nevertheless, a complicated and high‐cost focused ion beam micromachining process is entailed to fabricate the nanograting, which makes this strategy impractical for large‐scale application.

**Figure 12:**
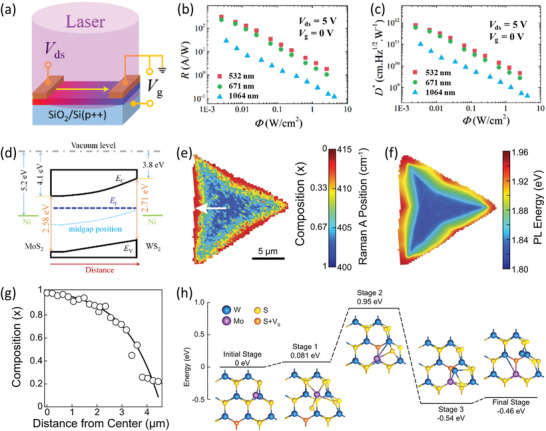
Graded 2DLM alloy for improved photosensitivity. a) Schematic diagram of a Mo_1−_ *_x_* W*_x_* S_2_ alloy photodetector with graded composition distribution. b,c) Responsivity and detectivity as a function of power density of incident light under illumination with different wavelengths. d) Band diagram of a Mo_1−_ *_x_* W*_x_* S_2_ alloy photodetector with graded composition distribution. *E*_C_, *E*_V_, and *E*_F_ represent CBM, VBM, and Fermi level, respectively.^[^ ^199^ ^]^ a–d) Reproduced with permission.^[^ ^199^ ^]^ Copyright 2019, American Chemical Society. e,f) Raman shift map of the A vibration mode and PL map of a CVD‐grown Mo_1−_ *_x_* W*_x_* S_2_ alloy domain with graded composition distribution. g) Composition as a function of position along the white arrow marked in (e). h) Schematic illustration of the diffusion‐exchange mechanism and the energies of the reaction steps for the inward diffusion of a Mo atom accompanied by the outward diffusion of a W atom and a S vacancy.^[^ ^230^ ^]^ e–h) Reproduced with permission.^[^ ^230^ ^]^ Copyright 2018, Nature Publishing Group.

To address these issues, in 2019, Yang et al. synthesized monolayer Mo_1−_ *_x_* W*_x_* S_2_ alloys with graded composition by a one‐step CVD approach and systematically explored its photoelectric characteristics (**Figure** **12**a).^[^ ^199^ ^]^ A wide span in composition (0.04 < *x* < 1) within a single domain is achieved by optimizing the positions of the precursors. As shown in Figure 12b,c, the effective wavelength range of the graded Mo_1−_ *_x_* W*_x_* S_2_ alloy photodetector covers 532 to 1064 nm. Under 532, 671, and 1064 nm illumination, the optimal responsivity reaches 298.4, 224.4, and 28.7 A W^−1^. These performance metrics are much superior to those of the homogeneous Mo_0.5_W_0.5_S_2_ alloy photodetector (*R* ≈ 5.8 A W^−1^)^[^ ^155^ ^]^ and the compositionally abrupt MoS_2_/WS_2_ heterojunction photodetectors (R ≈ 68 mA W^−1^–2.3 A W^−1^).^[^ ^229^, ^287^, ^288^ ^]^ In addition, the corresponding detectivity reaches 7.6 × 10^11^, 5.9 × 10^11^, and 1.0 × 10^11^ Jones, respectively. The excellent device performances are mainly attributed to the high‐efficiency separation of photocarriers across the whole photosensitive channel, which is enabled by the graded composition distribution. Specifically, MoS_2_ and WS_2_ bear a staggered type‐II band alignment, where the CBM and VBM of WS_2_ are both higher than those of MoS_2_, respectively. As a result, the graded element distribution of the Mo_1−_ *_x_* W*_x_* S_2_ alloy results in a gradient band offset, where the more the W atoms included, the higher the CBM and VBM are, as shown in Figure 12d. As a consequence, an omnipresent in‐plane driving force is generated across the whole photosensitive channel to efficiently promote the separation of photogenerated electron–hole pairs, thereby prolonging the lifetime of photocarriers and thus improving the photosensitivity. Following this success and working principle, Xu et al. developed high‐performance photodetectors based on vertically graded 2DLM alloy as well.^[^ ^289^ ^]^ In this study, graded MoS_2(1−_ *_x_*_)_Se_2_ *_x_* alloy with Se content increasing along the normal direction is employed as the photosensitive channel. Conforming to the gradient composition induced immanent out‐of‐plane built‐in electric field, which efficiently drives electrons and holes to transport to opposite directions, this device is operable under a self‐powered working mode, with an outstanding responsivity of 311 mA W^−1^. This value is significantly higher than those of the reported self‐powered photodetectors based on lateral MoS_2_/MoSe_2_ heterojunctions (*R* ≈ 18 mA W^−1^).^[^ ^290^ ^]^ In addition, this device achieves a satisfactory detectivity of ≈10^11^ Jones. Under a source‐drain bias of −0.5 V, the responsivity can be further boosted to 23.2, 191.5, and 26.2 A W^−1^ when excited by 405, 650, and 808 nm lasers, and the detectivity is at the magnitude of ≈10^12^ Jones. Moreover, the response/recovery time of the MoS_2(1−_ *_x_*_)_Se_2_ *_x_* photodetector is as short as 51 ms/51 ms. On the whole, these values also outperform those of pristine MoS_2_ (*R* ≈ 7.5 mA W^−1^, *τ*_rise_/*τ*_decay_ ≈ 50 ms/50 ms),^[^ ^291^ ^]^ MoSe_2_ (*R* ≈ 13 mA W^−1^, *τ*_rise_/*τ*_decay_ ≈ 60 ms/60 ms)^[^ ^292^ ^]^ photodetectors, the mosaic MoS_2_/MoSe_2_ heterojunction photodetector (*R* ≈ 1.3 A W^−1^, D* ≈ 2.6 × 10^11^ Jones, *τ*_rise_/*τ*_decay_ ≈ 0.6 s/0.5 s)^[^ ^293^ ^]^ and comparable to those of the *in‐situ* fabricated vertical MoS_2_/MoSe_2_ heterojunction photodetector (*R* ≈ 36 A W^−1^, D* ≈ 4.8 × 10^11^ Jones, *τ*_rise_/*τ*_decay_ ≈ 1.5 ms/3.5 ms).^[^ ^294^ ^]^ In general, these experimental findings have established that the composition gradient provides an additional degree of freedom to tailor the photoresponse of 2DLMs‐based photodetectors, without structure abruption as in the case of heterojunctions. Such strategy can ameliorate the photosensitivity of 2DLM photodetectors whilst circumventing the complicated device structures such as the split‐gate configuration as well as the labor‐intensive device fabrication process.^[^ ^295^ ^]^ By optimizing the bandgap offset and span, the device performances can be further improved.

Importantly, Bogaert et al. recently found that point defects played a vital role in the alloying of 2DLMs.^[^ ^230^ ^]^ As shown in Figure 12e–g, the graded Mo_1−_ *_x_* W*_x_* S_2_ alloy that span nearly the full composition range along a single domain is controllably synthesized through a diffusion‐based CVD method, that is, synthesis of defective WS_2_ skeleton followed by Mo incorporation in the subsequent step.

As shown in Figure 12h, density function theory indicates that Mo atoms easily diffuse inside with the assist of S vacancies, simultaneously rendering the outward diffusion of W atoms. Since this diffusion‐driven metal exchange process is dominated by S vacancy, the composition distribution can thus be readily controlled by tuning the vacancy distribution of the starting WS_2_ templates. Despite that only graded Mo_1−_ *_x_* W*_x_* S_2_ alloy is synthesized in this work, this strategy theoretically can be generally applicable across the entire breadth of 2DLM platforms. Thus far, strategies such as focused ion beam irradiation,^[^ ^296^ ^]^ e‐beam irradiation,^[^ ^297^ ^]^ plasma bombardment,^[^ ^298^ ^]^ and vacuum annealing^[^ ^299^ ^]^ have been developed to deliberately create defects in 2DLMs, which can be powerful tools for engineering the defect distribution toward on‐demand design of graded 2DLM alloys. Therefore, persistent efforts are expected to be devoted to the precise customization of the composition profiles of 2DLM alloys for advanced photodetectors in the future.

## 5 Conclusion and Outlook

As a whole, alloying can markedly expand the technological landscape of 2DLMs by introducing fresh blood with various exceptional features, distinguished from the corresponding host materials, via composition engineering whilst inheriting the communal advantages of vdW materials. In the past several years, 2DLM alloys have witnessed a prosperous development in both fundamental science and device applications. For example, alloys of 2DLMs with opposite charge‐carrier polarities can be exploited to implement ambipolar FETs.^[^ ^180^ ^]^ In addition, through rational design of energy band structure, alloying can effectively suppress deep‐level defect states, which gives rise to both ameliorated photosensitivity and expedited response rate of the corresponding 2DLM photodetectors.^[^ ^155^ ^]^ In a word, 2DLM alloys have markedly enriched the library of 2D materials and these fascinating members enable substantial property modulation toward on‐demand device design and applications. In this regard, 2DLM alloys promise fertile building blocks potentially complementing the shortcomings of the stoichiometric host materials. However, compared with S2DLMs, the research on 2DLM alloys is still in its infancy. In the wake of the deepening of 2DLM alloy research, a series of new issues head for researchers. Below, we highlight the ongoing challenges in this fast‐evolving domain, accompanied with the future opportunities.

### 5.1 Suppression of Competing Phase Segregation and Non‐Uniform Composition Distribution

Unintentional and unmanageable phase segregation as well as non‐uniform composition distribution are still in the rank of the most pivotal challenges plaguing the preparation and practical application of 2DLM alloys, especially for those consisted of host materials with significantly distinct structural and physical properties. For example, there is a miscibility gap (from 14% to 70% Te concentration) where phase separation easily occurs for MBE growth of 2D WSe_2−_ *_x_* Te*_x_* alloy.^[^ ^174^ ^]^ As a consequence, numerous interspersed semiconducting domains and metallic domains co‐exist in the MBE‐derived 2D WSe_2−_ *_x_* Te*_x_* alloy. The phase segregation and non‐uniform composition distribution seriously preclude the exploration and harness of the intrinsic physical properties of the nominal 2DLM alloys. In this regard, they can render considerable device­to­device variation, which is inconducive to practical application. Generally, the difficulty of producing high‐quality 2DLM alloys lies in the complexity and inadequate understanding of the phase diagrams of multicomponent layered materials, as well as, the inadequate reactions among multiple compositions. As for the former, theory calculations are efficient and powerful means to acquire the phase diagram and screen the thermodynamically stable 2DLM alloys.^[^ ^300^, ^301^ ^]^ Most recently, machine learning has been identified as another effective route for predicting the thermodynamic stability of 2DLMs as well.^[^ ^302^ ^]^ Therefore, machine learning‐driven design of 2DLM alloys represents another potentially feasible direction of this domain. As for the latter, increasing the synthesis temperature is an effective strategy to improve the composition uniformity as it can provide sufficient energy for interdiffusion and to surmount the reaction barriers.^[^ ^140^, ^303^ ^]^ However, it is to be concerned that this strategy may bring additional dilemmas such as increased thermal defect density.

### 5.2 Exploration of Untapped 2D Layered Material Alloys

Thus far, the research enthusiasms on 2DLM alloys have been mostly focused on the MX_2_ TMDC alloys, while the experimental realization of alloys based on other 2DLMs are relatively rare. This significantly limits the scope of property modulation enabled by alloy engineering. For example, there is still no report on the alloys of TDMCs and transition metal halides, although many of these members share a common stoichiometric formula (e.g., WSe_2_^[^ ^304^ ^]^ and NiI_2_^[^ ^305^ ^]^) and can be plausibly completely alloyed. In this regard, the diversity of 2DLMs and the variation of stoichiometric ratio of the constituent elements theoretically provide infinite schemes for the design and implementation of novel 2DLM alloys. Theoretical analysis is a potent thrust to develop and screen mysterious 2DLM alloys with prospective physical properties, which help to circumvent the unavailing material synthesis and characterization to a certain extent. For example, recently, Hemmat et al. have excavated 25 kinds of 2DLM alloys through the equilibrium temperature‐composition phase diagrams acquired by first‐principles calculations, and they have synthesized 12 of them by employing a single CVT approach.^[^ ^306^ ^]^

### 5.3 Exploration of Exotic Working Principles of 2D Layered Material Alloy Devices

Thus far, only a portion of studies on 2DLM alloys based electronic and optoelectronic devices have been explicitly elucidated. Many experimental advancements are still in need of in‐depth analysis.^[^ ^307^ ^]^ This is a serious oversight as the underneath physical origins of exceptional findings are fundamental to the design and implementation of future electronic and optoelectronic devices based on 2DLM alloys.

### 5.4 Alloys beyond Binary and Ternary Systems as well as Alloys beyond Layered Materials

Compared with binary and ternary alloys, 2DLM alloys with four or more elements bear more degrees of freedom for property modulation, enabling them theoretically hold more abundant physical properties. For example, the bandgap of ternary phosphorus chalcogenides lies in the range of 1.3–3.5 eV.^[^ ^308^ ^]^ Therefore, through rational alloying, photosensitive channels that exactly coincide with all wavelengths from ultraviolet to near‐infrared can be available. On the other hand, non‐layered 2D materials have been successfully synthesized through a series of approaches represented by vdW epitaxy, 2D template assisted topotactic transformation, space‐confined growth method, self‐limited growth method, etc.^[^ ^309^ ^]^ The incorporation of non‐layered 2D materials for alloying prominently enriches the building blocks toward the construction of 2D semiconductor alloys. For example, layered Bi_2_Te_3_,^[^ ^310^ ^]^ non‐layered In_2_S_3_,^[^ ^286^ ^]^ and In_2_Te_3_^[^ ^311^ ^]^ share a common stoichiometric formula (M_2_X_3_) but distinct crystal structures and physical properties, where Bi_2_Te_3_ is a topological insulator whereas In_2_S_3_ and In_2_Te_3_ are semiconductors. Therefore, the alloys based on them are appealing subjects for the research efforts in the future.

### 5.5 Developing 2D Layered Material Alloys for Light‐Emitting Diode Application

Light‐emitting diodes (LEDs) represent devices that can enable photon emission by driving electrons and holes to recombine with the assist of an external electric field.^[^ ^312^, ^313^ ^]^ Due to the favorable bandgap, outstanding electrostatic tunability and excellent mechanical properties, 2DLMs have demonstrated grand potential in LEDs as well.^[^ ^314^, ^315^, ^316^ ^]^ Nevertheless, there are still several issues to be addressed prior to the extensive utilization of 2DLMs in LED. The first one is that the active light‐emitting materials should hold a direct bandgap, which enables phonon‐free electron transition, thereby realizing high‐efficiency light emission. In addition, the active materials are anticipated to bear continuous bandgap values to meet the light‐emission requirements of different wavelengths. Moreover, there need to be 2D building blocks with various charge‐carrier polarities to enable the construction of p‐n junctions. Unfortunately, a substantial portion of 2DLMs are indirect bandgap semiconductors. For example, the most commonly studied group VIB TMDCs exhibit direct bandgap only in the monolayer form. Few‐layer and multilayer group VIB TMDCs are indirect bandgap semiconductors, which hinders their application in LEDs. In addition, the bandgap distribution of S2DLMs is relatively discrete, and they thus cannot fully meet the requirements of light emission with diverse wavelengths. Furthermore, most 2DLMs exhibit n‐type conductance, which hinders the design and construction of p‐n junctions. Fortunately, the latest investigations have suggested that alloying can effectively regulate the band structure of 2DLMs. For example, Ernandes et al. found that WS_0.4_Se_1.6_ alloy exhibited a strong immunity to PL quenching with increasing thickness as compared to binary WS_2_ and WSe_2_.^[^ ^88^ ^]^ Sainbileg et al. have revealed that Ni substitution on the Sn sites of SnS_2_ generates new band lines at the bottom of the conduction band due to strong orbital hybridization with states of its nearby sulfur atoms, which results in intriguing indirect‐to‐direct bandgap transition.^[^ ^317^ ^]^ On the other hand, as demonstrated in previous studies, both bandgap value and charge‐carrier polarity of 2DLMs can be markedly modulated by alloying.^[^ ^86^, ^180^ ^]^ Therefore, 2DLM alloys hold enormous potential in LED applications, and further exploration is urgent need in the coming future.

As a concluding remark, alloying has served as a variable and flexible avenue to tailor the attributes of 2DLMs with a high degree of tunability, bringing a variety of exotic physical properties and applications beyond the fundamental limits of S2DLMs. Therefore, 2DLM alloys have ushered the new frontier of materials science and they are important contenders for constructing electronic and optoelectronic devices. It is envisioned that after overcoming the above‐mentioned predicaments, 2DLM alloys will be an indispensable complement to the existing S2DLMs and play a vital role in the forthcoming breakthroughs of semiconductor technology nodes.

## Conflict of Interest

The authors declare no conflict of interest.
